# Integrated electrical modeling of circulating tumor cells for enhanced dielectrophoretic trapping and electroporation

**DOI:** 10.1038/s41598-026-45747-z

**Published:** 2026-04-12

**Authors:** Sameh Sherif, Yehya H. Ghallab, Yehea Ismail

**Affiliations:** 1 School of Electronics, Communication and Computer Engineering, Egypt-Japan University of Science and Technology (E-JUST), New Borg El Arab, Egypt; 2 Biomedical Engineering Department, Capital University (Formerly Helwan University), Cairo, Egypt; 3 Center of Nanoelectronics and Devices (CND), Zewail City of Science and Technology, The American University in Cairo (AUC), Giza, Egypt

**Keywords:** Biological techniques, Biophysics, Cancer, Cell biology, Engineering

## Abstract

This study presents a comprehensive computational and experimental investigation of the integrated dielectrophoresis (DEP) and electroporation framework for the selective manipulation and controlled treatment of circulating tumor cells (CTCs), white blood cells (WBCs), and platelets (PLTs) within a unified microfluidic platform. A multiphysics mathematical model, implemented in COMSOL Multiphysics, is developed to simulate the complete sequence of DEP-driven cell trapping followed by pulsed electric field electroporation, capturing the dynamic processes of membrane charging, pore nucleation, growth, and resealing under short-duration 2 µs electric pulses. The key electroporation parameters, including transmembrane potential, pore radius, pore density, and membrane conductivity, are systematically characterized for each cell type to define cell-specific optimal pulse protocols that maximize treatment efficacy while preserving cell viability and minimizing thermal effects. The DEP mechanism provides stable spatial confinement of target cells between electrode pairs, enabling precise and reproducible exposure to calibrated electric fields with reduced off-target perturbations. Comparative computational analysis across the three cell types reveals that the requisite electric field strength must be tailored to cell dimensions, with 1–4 kV/cm identified as appropriate for CTCs and WBCs and 10–40 kV/cm required for platelets owing to their substantially smaller diameter and correspondingly higher membrane charging threshold. The simulation results demonstrate spatially heterogeneous pore dynamics and electric displacement field distributions across the cell membrane, with the most pronounced effects concentrated at the hyperpolarized pole, underscoring the critical influence of cell geometry and electric field distribution on electroporation outcomes. To experimentally validate the computational predictions, a dedicated microfluidic platform integrating microfabricated electrode arrays with real-time impedance sensing and optical monitoring was developed and applied to THP-1 monocytic cells as a representative model system. The device comprises a central impedance sensor defining the active sensing zone and surrounding focusing electrodes with lateral dimensions of $L = w = 600$ µm and an inter-electrode spacing of 200 µm, electrically routed through via connections for independent excitation and measurement. Impedance spectroscopy was performed over a frequency range of $10^{3}$–$10^{6}$ Hz under applied voltages from 1 to 25 V across the 200 µm sensing gap. The impedance magnitude (|*Z*|) exhibited a clear monotonic decrease with increasing applied voltage, with the most pronounced reductions observed in the mid-frequency range (10–100 kHz), confirming enhanced membrane permeability and increased effective conductivity of the cell suspension under stronger electric fields. The reactive impedance component ($X_s$) demonstrated progressive suppression of negative reactance with increasing voltage, indicating systematic loss of membrane capacitive behavior, while the series resistance ($R_s$) decreased and stabilized at higher voltages, reflecting a transition from capacitance-dominated to conductivity-dominated electrical transport.

## Introduction

Electroporation^1^ is a noninvasive technique to examine the behavior, separation, analysis, and treatment of different cells and cancer cells, such as circulating tumor cells (CTCs) and other blood cells. It enhances the accuracy and efficiency of cancer research procedures and therapeutic approaches. Electroporation enables controlled ingress and egress of materials or molecules into and out of cells by rendering cell membranes permeable using short electric pulses^1,2^. Several studies have investigated the impact of electroporation therapy methods compared to conventional approaches. Electroporation microfluidic chip has demonstrated numerous functions focused on cell analysis and research^3,4^.

CTCs^5^ are rare cells that come from tumors that are primary and are essential for finding, treating, and monitoring the cancer^5^. Their selective examination is vital for early cancer detection as well as customized therapy, according to their unique morphological and behavioral characteristics relative to other components of the blood. Dielectrophoresis (DEP)^6,7^ and other microfluidic systems ^8,9^ effectively isolate and manipulate circulating tumor cells (CTCs)^10^, enabling real-time, minimally invasive drug delivery and intracellular component extraction,^11,12^. This ability for functional profiling of living cells, which leads to more effective ways to monitor and classify cancers.

Electroporation is vital for targeted cancer treatments, especially electrochemotherapy, in addition to capturing and moving cells. Chemotherapy agents^13,14^ that usually cannot penetrate through cell membranes may penetrate through CTCs by electroporation, which temporarily opens cell membranes ^15^. This allows precise drug targeting in specific regions with minimal adverse effects. The procedure can be either irreversible, meaning that it will destroy tumor cells for a long time, or reversible, meaning that it will only change the genes or molecules for a short time. Electroporation also causes immunogenic cell death (ICD)^16^, which releases damage-associated molecular patterns that boost the immune response and make immunotherapy more effective ^16^.

Advanced research has focused on creating microfluidic and electroporation systems^17^enabling rapid and controllable cell manipulation with minimal viability damage. These systems ensure spatial and temporal regulation of electroporation parameters.

In another example, the multifunctional branched nanostraw-electroporation system^18^ showed that it could extract cytosolic 1 in real time and deliver biomolecules into single CTCs. These types of technological and technique advances present new ways or methodolgies to deliver drugs with precision and study cancer cells in depth.

Microfluidic chips^19,20^ have changed the way CTCs detecting and treatment protocols are applied by making it applicable to extract rare cancer cells from blood samples with high efficiency and selectivity rates. Conventional, or the previous, methods for isolating CTCs used physical filtration or size-based separation with mesh microfilters, micropost filters, or micromachined hole arrays. These techniques build on the advantage of the reality that CTCs are various in shape and size than other blood components.

Microellipse filters, for example, are exceptionally well at capturing cells while keeping tumor cells alive or maintain the viability^21^. Even with these improvements, it’s still hard to handle clinically significant blood volumes and get cells for molecular tests.

The microfluidic platforms use dielectric and size-based properties to combine dielectric, electrostatic, and immunological methods to improve CTC purity. Applying alternating current (AC) waveforms as the excitation signal to microelectrodes creates DEP forces that are based on CTC dielectric parameters, or the response for the applied electric field is function in the dielectric features of CTC.

This makes it possible to keep cells in the microchannel focal region without changing their properties or needing to label them. Such integrated systems show high recovery rates and purity in clinical samples, which helps with point-of-care cancer diagnostics and personalized treatment by allowing both physical and biochemical analysis of CTCs on-chip at the same time.

The microfluidic chips additionally utilize combinations multiple features for various analysis such as nanostructured arrays for single-cell analysis, hydrodynamic flow separation, and antibody-mediated bead capture. the advantages of these chips are label-free separation, antigen-independent isolation, and nanodroplet arrays in microreactors improve and enhance the capture efficiency and make it applicable to profile enzyme activity and protein expression following the detection stage. Using several isolation methods at the same time greatly increases the purity and viability of CTCs, which leads to better data for clinical testing.

Dielectrophoresis (DEP)^22^ remains a potent, label-free technique for cell separation based on variations in cellular membrane permittivity and conductivity. DEP-based microfluidic devices require optimized electrode geometry and precise electric field control for maximum selectivity and throughput^23,24^. Wireless bipolar ^25^electrodes and optically induced DEP have achieved rapid single-cell capture and large blood volume processing^26^. However, the dielectric features, such as blood conductivity, osmotic stress^27^, and device configuration, consider high influence and impact on the performance and required to be optimized for clinical translation^28^.

Electroporation is integrated into next-generation microfluidic chips and platforms for therapeutic delivery, targeted CTC analysis, and selective cellular ablation, addressing cancer treatment challenges while limited and decreasing collateral damage to healthy cells.

The pore radius formed during electroporation is a critical determinant of molecular transport across membranes^29,30^. Electroporation creates a distribution of pores varying in size: small pores facilitate ion and small molecule passage, while larger pores allow protein and macromolecule ingress^31^. Pulse duration and field intensity influence pore size and lifetime Larger pores are transient and reseal quickly, while smaller pores persist longer. This selective permeability underpins reversible or irreversible electroporation, depending on therapeutic intent.

Membrane conductivity also impacts permeability, survival, and electroporation efficiency^32^. Higher membrane conductivity, driven by larger pore formation, results in greater ion transfer across the membrane^33^. The external medium’s conductivity similarly determines the required field strength for effective electroporation^34^. Therefore, modulation of cell polarization and medium conductivity is crucial for safe and accurate cellular manipulation.

Electroporation pore formation is driven by transmembrane potential, amplified by an applied electric field. When this potential surpasses a threshold (250 mV–1 V)^35^, pores form, allowing material exchange. The magnitude of induced potential depends on cellular dielectric characteristics, field strength, membrane properties, and pulse duration^1^. Experimental and theoretical models confirm that controlled adjustment of these factors ensures reproducible and safe electroporation outcomes.

Pore resealing kinetics^36^, membrane area affected, and associated structural and functional modifications further characterize the electroporation response^37^. In reversible electroporation, rapid resealing preserves cellular integrity. By tuning pore size, distribution, conductivity, and transmembrane potential, modern electroporation methods selectively target CTCs with minimal impact on healthy surrounding cells^38^. Enhancing these parameters is critical for advancing electroporation-based therapeutic delivery, CTC sorting, and precision oncology technologies.

In both in vitro and in vivo settings, electroporation has gained significant interest as a biophysical tool for tumor therapy. High-voltage pulses transiently increase membrane permeability, augmenting cellular uptake of therapeutic molecules^39^. The success of electroporation depends heavily on pulse duration and intensity: high field strength with micro- to millisecond pulses may yield reversible or irreversible effects. Conventional pulses typically last longer than the characteristic charging time of a cell ( 100 ns for a 10 $\upmu$m radius cell), thus primarily affecting the plasma membrane. By contrast, nanosecond pulses ($10^6{-}10^7$ V/m) better match membrane charging dynamics, with minimal thermal impact^1,40,41^.

The primary goal of this study is to investigate cellular responses to electroporation across varying electric field intensities and pulse durations. We analyze transmembrane potential distribution at hyperpolarized and depolarized poles, as well as pore formation and evolution over time. Furthermore, the resealing rate under distinct stimulation conditions is evaluated, considering that neuronal membranes may require up to 20 seconds to restore resting potential ( 75 mV) post-stimulation.

This work examines how millisecond and nanosecond pulses differently affect membrane permeability and conductivity across cancer cell types to establish optimized electroporation conditions for diverse electrophysiological profiles.

The main goal is to create selective electroporation therapies that can identify the difference between platelets (PLTs), white blood cells (WBCs), and CTCs. This will assist in making sure that the therapies are as effective as possible while causing minimal damage to healthy cells as attainable. Dielectrophoresis (DEP) is an additionally method for selectively capturing cells and aligning them before electroporation phase. DEP utilizes the advantage of how cells become polarized when they are in fluctuating or nonuniform electric fields ^42^. This enabling the controlling of relocated WBCs, CTCs, and other blood-derived cells instead of using biochemical labeling. In microfluidic systems, DEP forces move target cells to a region of convergence (ROC) before electroporation begins. This makes sure that all cells are exposed to the same electrical pulses. The control in the field strengths and pulse durations required to keep CTCs alive. As a result, DEP-assisted trapping makes electroporation more accurate, selective, and repeatable for both therapeutic and analytical purposes. DEP trapping uses differences in the size, shape, and dielectric properties of cell membranes to tell CTCs apart from other blood cells. DEP devices create selective trapping forces by changing the frequency and amplitude of the applied electric field. This allows smaller platelets and red blood cells (RBCs) to move through whereas maintaining larger, more polarizable CTCs. This process of enrichment makes it possible to get rare CTCs from complex samples with little contamination, making sure that the preparation is free of contaminants for the next step of electroporation. Combining DEP trapping with electroporation also makes it possible to continuously process a lot of cells in closed-loop microfluidic systems, which minimizes contamination and material loss. The DEP–electroporation hybrid method is perfect for clinical precision oncology because it provides quick diagnoses, reliable results, and low sample requirements for thorough cancer cell profiling and targeted therapy.

Finally, this paper is structured as follows: “Proposed mathematical model” outlines the mathematical modeling and governing equations. “Simulation results” presents the simulation results focusing on electroporation parameters, while “The appropriate condition for CTC cells” discusses their biomedical implications for targeted cancer therapy.

## Proposed mathematical model

**Fig. 1:**
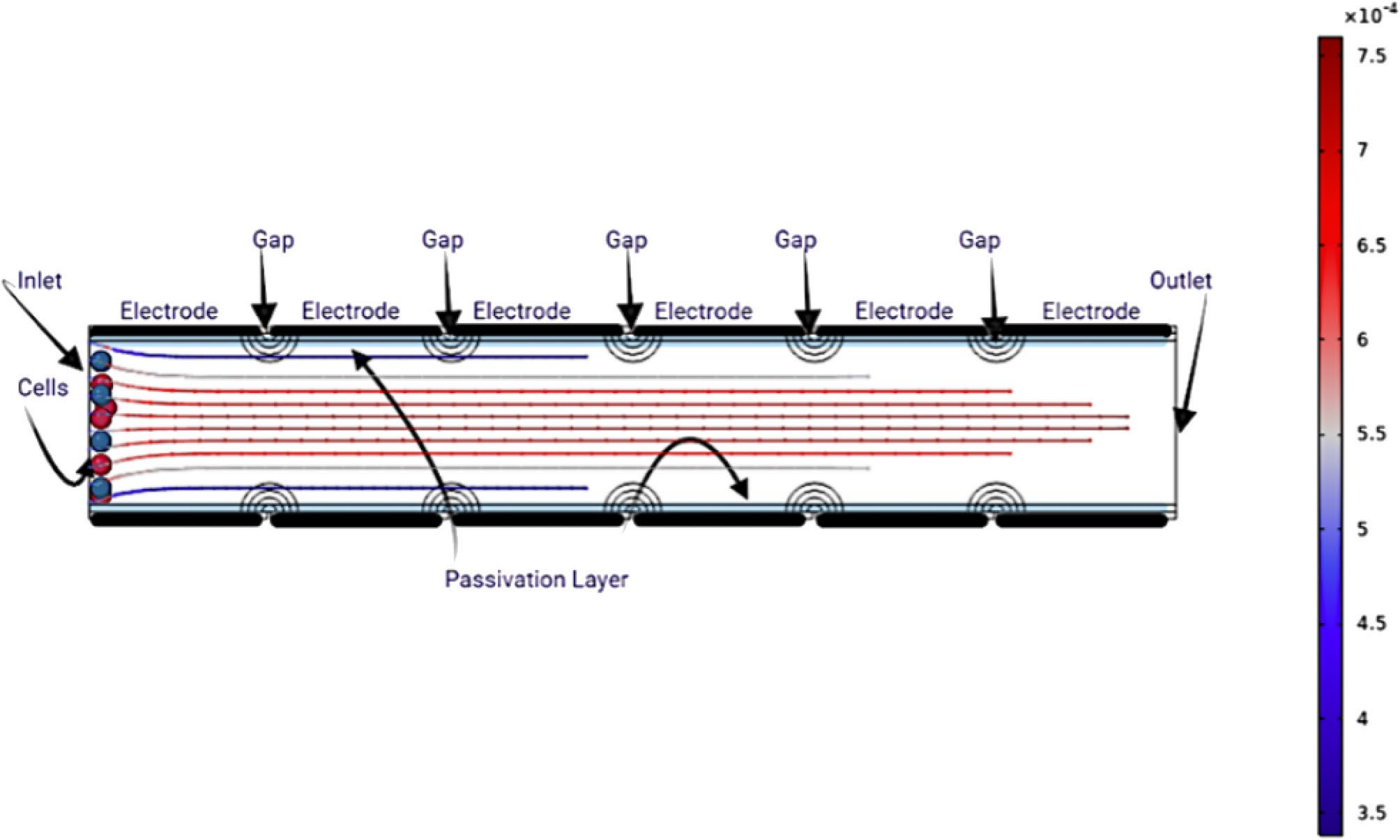
Schematic showing the steady-state flow of a CTC-containing medium in the microchannel without DEP force influence.

Trapping and cell focusing phenomena are two key mechanisms that describe the flow behavior of circulating tumor cells (CTCs) inserted into a microfluidic channel. The medium containing CTCs flows under steady-state conditions, as illustrated in Fig. 1, where no external electric field is applied hence, no dielectrophoretic (DEP) force acts on the cells. Under these circumstances, the cluster of cells travels from the channel inlet to the outlet without external trapping influences. In the proposed model, the electrodes incorporate a passivation layer to enhance cell viability, as discussed in^43^.

**Fig. 2:**
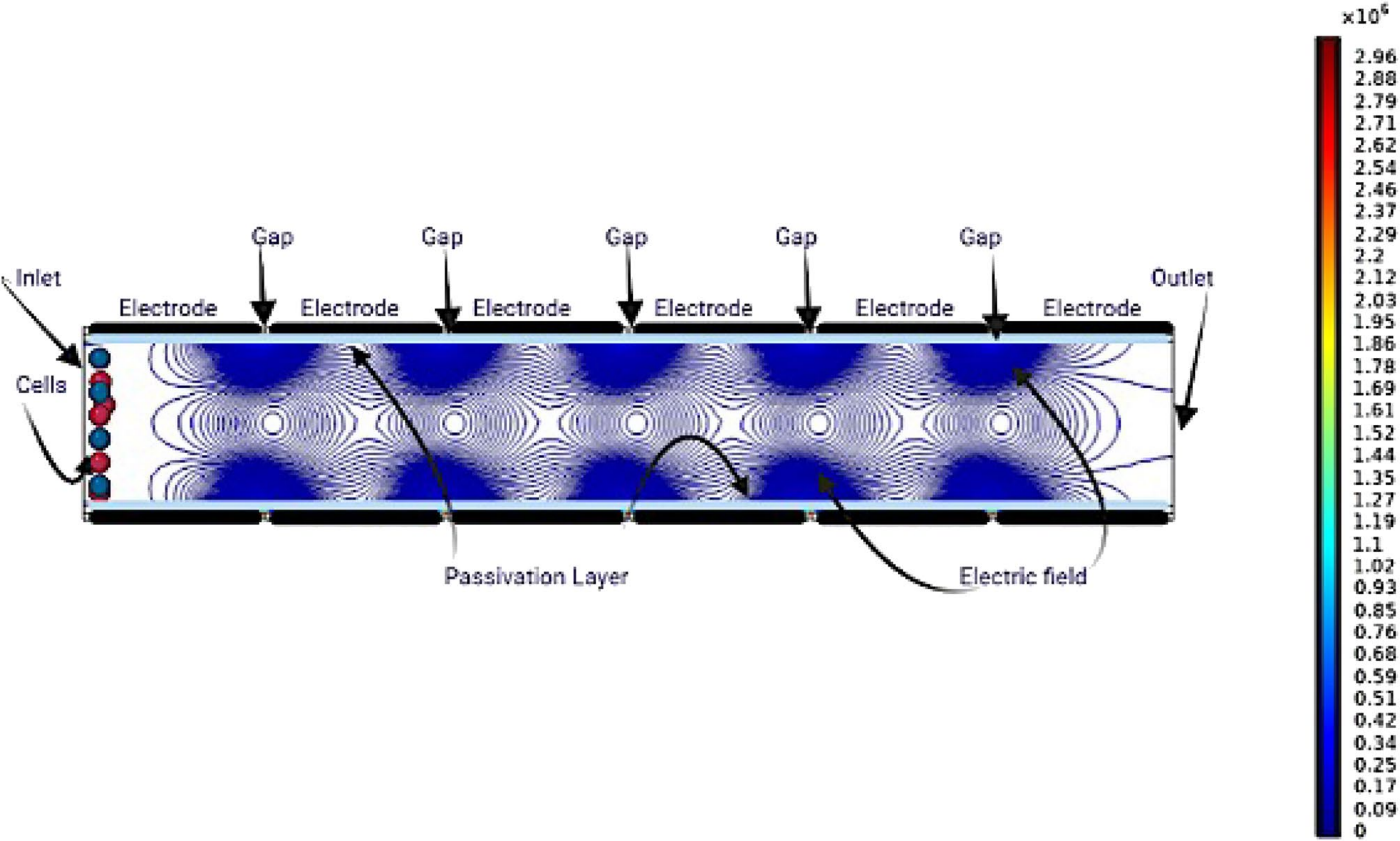
Electric field norm distribution map illustrating the non-uniform AC electric field applied across channel electrodes.

**Fig. 3:**
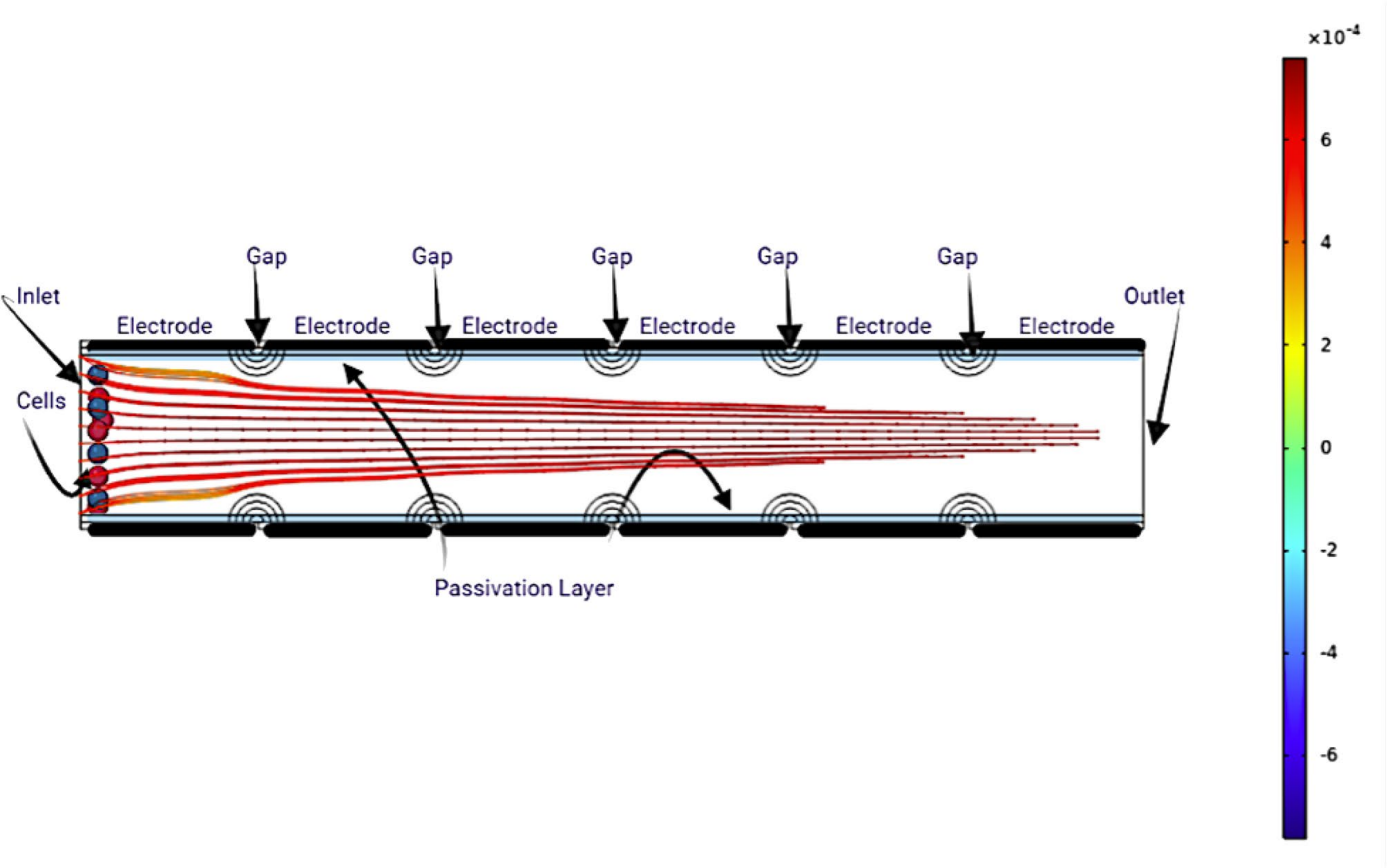
CTC streamlines showing cell focusing behavior under specific DEP conditions.

When an alternating current (AC) electric field is applied, as shown in Figs. 2 and 3, the voltage distribution is adjusted to satisfy DEP force criteria, allowing effective CTC trapping. The non-uniform electric field distribution arises from the sinusoidal voltages applied at each electrode, each differing by a controlled phase shift. This field variation produces localized regions of high and low intensity, which govern cell motion and focusing.

**Fig. 4:**
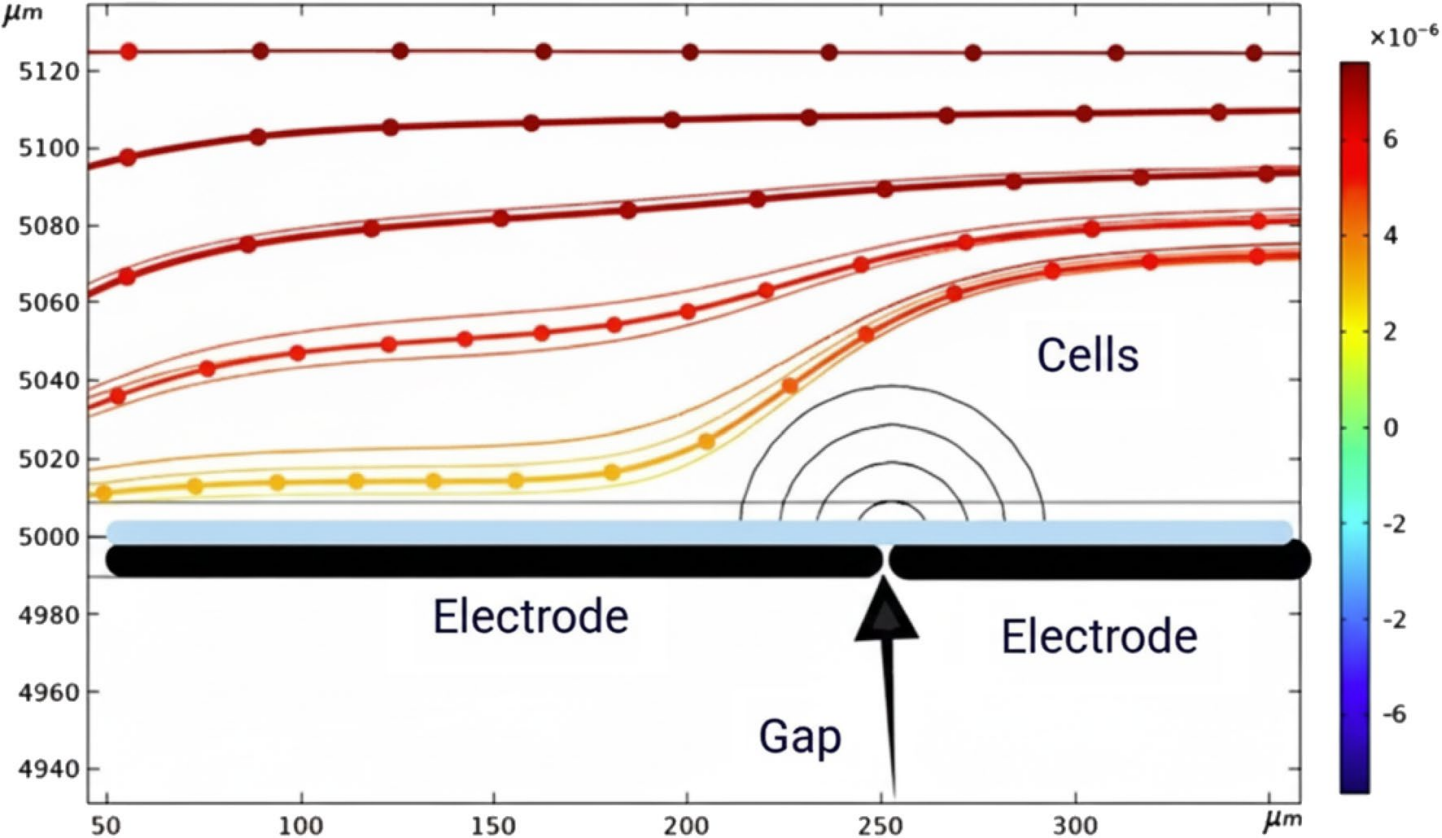
Cell displacement away from high-field zones under negative dielectrophoresis, illustrating controlled positioning.

As illustrated in Fig. 4, CTCs experience repulsion from areas of high electric field intensity, causing them to align along equilibrium streamlines where the DEP forces are balanced.

**Fig. 5:**
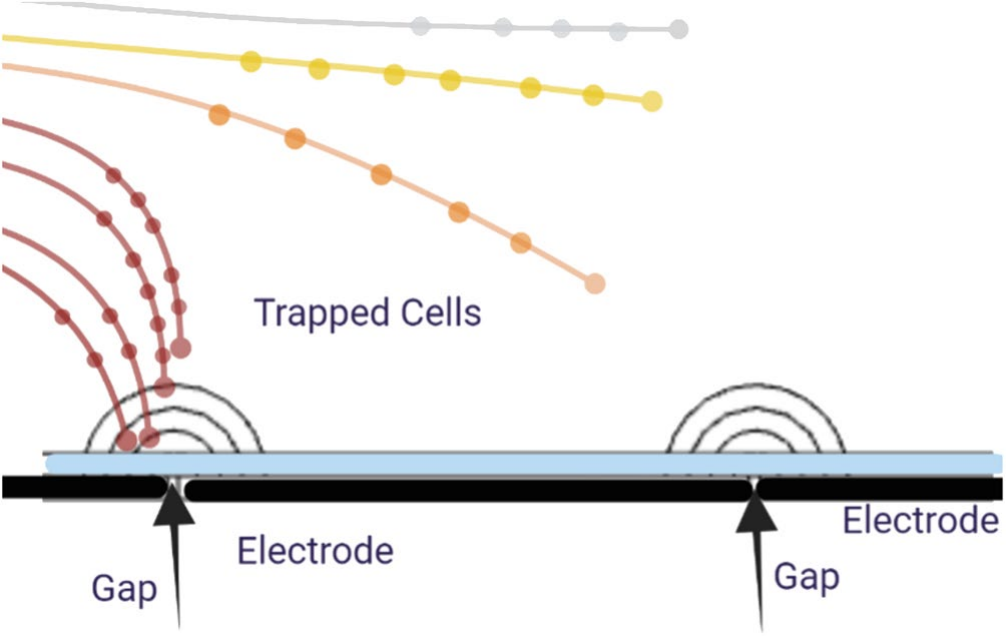
Trapping of CTCs between electrodes caused by spatially controlled DEP forces, ensuring optimal alignment in the electroporation zone.

This phenomenon facilitates the accurate manipulation and isolation of circulating tumor cells (CTCs) within a microfluidic setup. With this level of control, the dielectrophoretic trapping technique can assist when hybrid cells form through electrofusion, such as between immune and tumor cells. This makes it beneficial for both diagnosis and treatment. As shown in Fig. 5, this study systematically uses both positive and negative DEP ideas to control where cells are in the electroporation area. DEP offers a noninvasive method to position cells in designated areas between electrodes, guaranteeing optimal exposure during electroporation pulses. By adjusting the DEP signal parameters, cells can be moved closer to or farther away from the high-field regions and detection areas. This provides high spatial precision over their spatial distribution. This sort of controlled positioning makes personalized electroporation protocols much more effective and easier to repeat. It also helps with targeted membrane permeabilization while keeping cells alive. Manipulating cells using DEP is a key part of the proposed method. It allows for precise transport and localization of cells within the electroporation chamber, which leads to the best possible therapeutic results. This study builds on earlier research that showed how DEP can accurately control and change how cells behave in microfluidic systems. This demonstrates further potential for DEP to be in clinical microengineering.

### Mathematical model

**Fig. 6:**
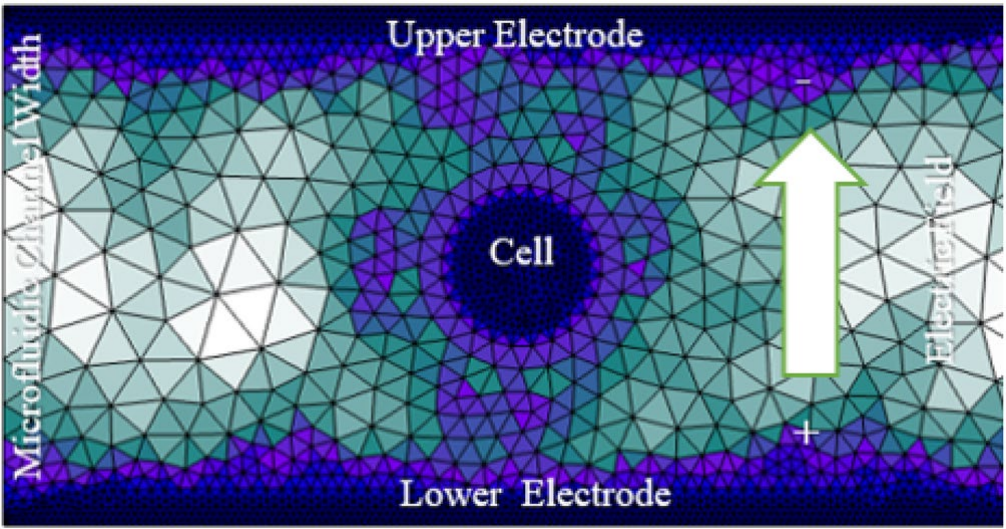
Schematic of the proposed microfluidic system for analyzing electroporation dynamics. The color gradient defines the free triangular mesh with minimum and maximum element sizes of $1~\upmu$m and $6~\upmu$m, respectively. The complete mesh includes 4526 domain elements and 642 boundary elements.

Following the completion of the dielectrophoresis (DEP)-based cell trapping process, the electroporation parameters of the targeted breast cancer cells were analyzed. The simulation setup confines the captured cell inside a microchannel, as illustrated in Fig. 6, and subjects it to alternating electric pulses with specific durations and intensities. Two sensing electrodes, one connected to the source and the other to ground, are embedded within the system; both are $100~\upmu$m wide and are separated by $50~\upmu$m. These electrodes deliver controlled electric fields for cell stimulation. The Heaviside function (*H*(*x*)), defined as the integral of the Dirac delta function $\delta (s)$, is employed as the excitation signal applied to the electrodes:

1 $$\begin{aligned} H(x) = \int _{-\infty }^{x} \delta (s) \, ds. \end{aligned}$$

This function enables sharp ^42^ between electric field states and simulates pulse onset accurately^1,2^. The stimulus consists of two distinct pulse lengths nanoseconds and milliseconds providing a systematic framework for studying the cellular response across different electroporation regimes^1,4^.

**Table 1:** Electrical properties of various cell types adopted from literature^44^.

Parameter	CTC	WBC	PLT
Cell diameter ($\upmu$m)	15	12	1.8
Cell membrane thickness (nm)	7	7	8
Cytoplasmic conductivity (S/m)	1.0	0.18	0.25
Membrane conductivity (S/m)	$9\times 10^{-7}$	$9\times 10^{-6}$	$1\times 10^{-6}$
Cell relative permittivity	50	80	50
Membrane relative permittivity	12.5	10	6

### Mathematical equations

The mathematical formulation is based on the electric potential *V*, determined using the *Electric Currents* module of COMSOL Multiphysics in a time-dependent mode^42^. The mathematical equation is expressed as:

2 $$\begin{aligned} -\nabla (\sigma _i \nabla V) - \nabla \frac{\partial (\varepsilon _i \nabla V)}{\partial t} = 0, \end{aligned}$$

where $\sigma _i$ and $\varepsilon _i$ denote electrical conductivity and permittivity, respectively. In this model, the circulating tumor cells (CTCs) are treated as spherical bodies composed of a resistive cytoplasmic interior surrounded by a capacitive membrane^1^.

#### Transmembrane potential formation

When exposed to an external electric field of strength *E*, polarization of the CTC membrane induces a transmembrane potential $U_m$^1,45^:

3 $$\begin{aligned} U_m = \frac{3ER\cos \theta }{2}, \end{aligned}$$

where *R* is the CTC radius, and $\theta$ is the angle between the electric field vector and the point on the cell surface. The induced transmembrane voltage (ITV), critical for membrane permeabilization^1^, is the difference between the intracellular and extracellular potentials:

4 $$\begin{aligned} ITV = V_\text {in} - V_\text {out}. \end{aligned}$$

#### Membrane current density

The current density ${\bf J}$ across the membrane, governed by distributed impedance boundary conditions, is described as^4^:

5 $$\begin{aligned} {\bf n} \cdot {\bf J} = \frac{\sigma _m}{d_m}(V - V_\text {ref}) + \frac{\varepsilon _m}{d_m} \left( \frac{\partial V}{\partial t} - \frac{\partial V_\text {ref}}{\partial t} \right) , \end{aligned}$$

where $\sigma _m$, $\varepsilon _m$, and $d_m$ represent the membrane conductivity, permittivity, and thickness, respectively; ${\bf n}$ is the outward unit normal vector.

#### Pore formation and electroporation dynamics

The rate of pore formation *N*(*t*) within the membrane is a function of the local ITV, described by a differential relation^1,46^:

6 $$\begin{aligned} \frac{\partial N}{\partial t} = \alpha \exp \left[ \left( \frac{ITV}{V_{ep}} \right) ^2 \right] \left[ 1 - \frac{N}{N_0} \exp \left( -q \left( \frac{ITV}{V_{ep}} \right) ^2 \right) \right] , \end{aligned}$$

where $\alpha$ is the pore creation constant, $N_0$ is the maximum pore density, *q* is a sealing coefficient, and $V_{ep}$ is the characteristic electroporation voltage. The increase in membrane conductivity due to pore formation ($\sigma _{ep}$) is given by^30,40,46^:

7 $$\begin{aligned} \sigma _{ep} = N \left( \frac{2\pi r_p^2 \sigma _p d_m}{\pi r_p + 2d_m} \right) , \end{aligned}$$

where $r_p$ and $\sigma _p$ denote the pore radius and pore conductivity, respectively. The distribution and stability of these pores determine the electroporation efficiency and reversibility^1,32^.

### Dielectrophoresis (DEP) force modeling

Dielectrophoretic manipulation offers non-invasive methods for CTC trapping, separation, and transport within microfluidic domains^11,12,47^. The time-averaged DEP force acting on a spherical cell of radius *R*, suspended in a fluid of permittivity $\varepsilon _f$, is expressed as^48^

8 $$\begin{aligned} {\bf F}_{DEP} = 2\pi \varepsilon _f R^3 \text {Re}[K_w] \nabla E^2, \end{aligned}$$

where $\text {Re}[K_w]$ is the real part of the Clausius–Mossotti (CM) factor, which depends on the cell’s dielectric properties and the frequency of the applied electric field.

#### Traveling-wave dielectrophoresis (twDEP)

For efficient cell transport in microchannels, traveling-wave dielectrophoresis (twDEP) is employed^49,50^. The CM factor is defined as:

9 $$\begin{aligned} K_w = \frac{\varepsilon _p^* - \varepsilon _f^*}{\varepsilon _p^* + 2\varepsilon _f^*}, \end{aligned}$$

where complex permittivities are given by:

10 $$\begin{aligned} \varepsilon _p^* = \varepsilon _p - \frac{i\sigma _p}{\omega }, \qquad \varepsilon _f^* = \varepsilon _f - \frac{i\sigma _f}{\omega }, \end{aligned}$$

with $\varepsilon _p$ and $\varepsilon _f$ representing the permittivities, and $\sigma _p$, $\sigma _f$ the conductivities of the cell and medium, respectively. $\omega$ is the angular frequency of the applied field.

The total twDEP force is given by^50^:

11 $$\begin{aligned} {\bf F}_{\text {trav-DEP}} = 2\pi \varepsilon _0 \varepsilon _m R^3 \left[ \text {Re}[K_w] \nabla E^2 + 2\,\text {Im}[K_w] \nabla \times (\mathbf {E_I} \times \mathbf {E_R}) \right] , \end{aligned}$$

where $\varepsilon _0$ is the free-space permittivity, $\varepsilon _m$ is the relative permittivity of the medium, and ${\bf E} = \mathbf {E_R} + j\mathbf {E_I}$ is the complex electric field vector. The imaginary component $\text {Im}[K_w]$ governs the motion along the traveling wave direction.

This comprehensive model enables coupled analysis of DEP-based CTC manipulation and electroporation-driven membrane permeability, providing critical insights into electric field interactions within microfluidic bioengineering systems^3,12,18^.

## Simulation results

**Fig. 7:**
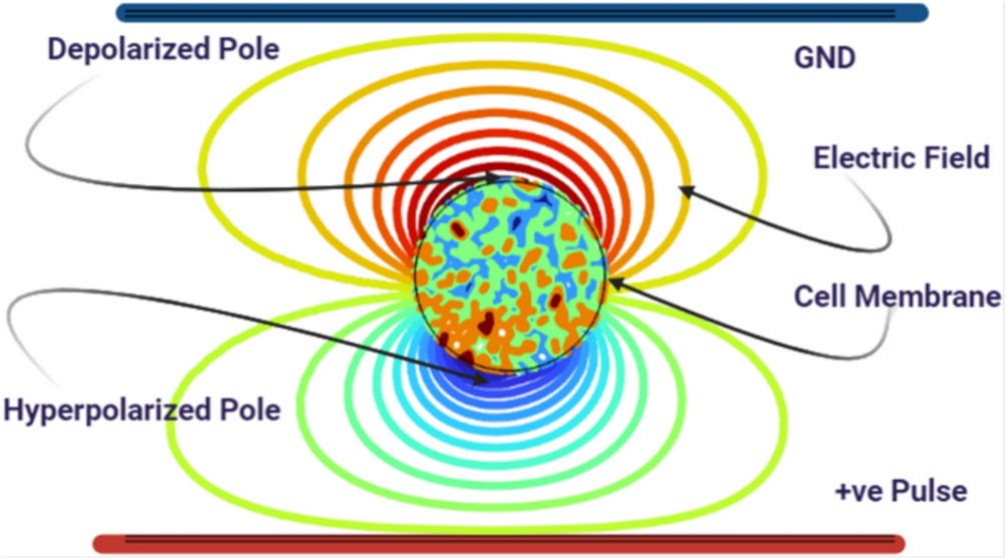
Schematic of a spherical cell considered in this study, showing the two main poles relative to the applied electric field.

In order to achieve selective targeting based on membrane electrical properties, the analysis focuses on the extracted features at the hyperpolarized and depolarized poles (Fig. 7), which are the two main membrane poles. Although there are only slight physical differences between circulating tumor cells (CTCs), platelets (PLTs), and white blood cells (WBCs), the substantial variations in membrane capacitance and conductivity listed in the parameter table provide a solid foundation for selective electroporation.

The goal of this research is to develop an optimal protocol that effectively treats and manipulates each cell type by exploiting their unique electrophysiological characteristics. The technique aims to optimize treatment effectiveness while minimizing damage to non-target cells by precisely adjusting pulse parameters such as transmembrane potential, pore radius, and membrane conductivity for each individual cell. This allows for targeted therapeutic interventions for CTCs, PLTs, and WBCs.

## The appropriate condition for CTC cells

This study focuses on the excitation of circulating tumor cells (CTCs) using pulse durations that align with their membrane charging characteristics. A pulse duration of $2~\mu \textrm{s}$ was selected to effectively interact with the typical charging time of CTC membranes.the selection of a short pulse duration (2 $\upmu$s) is grounded in well-established electroporation theory and supported by prior experimental and computational studies demonstrating that membrane charging and pore nucleation occur on sub-microsecond to microsecond time scales, whereas appreciable Joule heating requires substantially longer energy deposition times ^51^. The applied electric field strength varies within the range $1{:}1{:}4~\mathrm {kV/cm}$ to evaluate the cells’ electroporation response under different intensities. These parameters are designed to optimize membrane permeabilization while preserving cell viability, thereby enabling selective targeting and manipulation of CTCs.

### Transmembrane potential dynamics

**Fig. 8:**
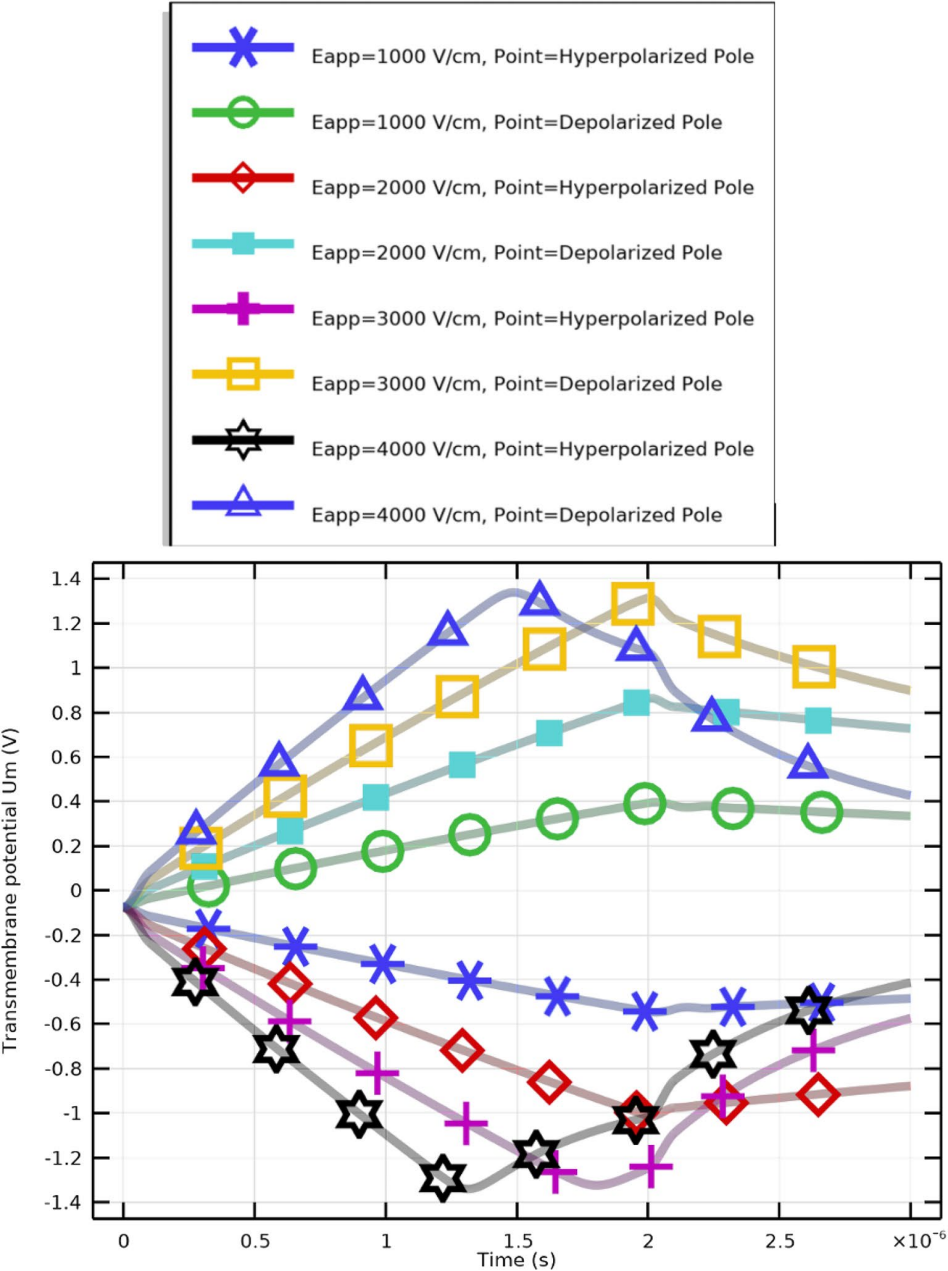
Transmembrane potential at the hyperpolarized and depolarized poles of CTCs under electric fields between 1 and 4 kV/cm.

Using the same categories of electric field strength, this section demonstrates how CTCs respond to an alternative excitation signal. The selection of this excitation is based on the matching of the pulse with the cell membrane charging time. As shown in Fig. 8, a longer pulse duration affects the transmembrane potential profile, enabling the critical transmembrane potential to be reached with a lower electric field.

Three main regions can be distinguished in the transmembrane potential distribution (Fig. 8):

A. Region A: charging time ($T = 1.0705\times 10^{-6}~\textrm{s}$)
B. Region B: pore nucleation ($2.0438\times 10^{-6}~\textrm{s}> T > 1.0705\times 10^{-6}~\textrm{s}$)
C. Region C: pore evolution ($T > 2.0438\times 10^{-6}~\textrm{s}$)

Application of an electric field initiates the charging phase, which ends once the membrane’s first pore forms. While pore nucleation occurs rapidly when the transmembrane potential exceeds approximately $\pm 1~\textrm{V}$, pore evolution progresses more slowly. Notably, as membrane leakiness increases, the charging time decreases. CTCs require more time to charge under weaker field excitations (Fig. 8).

It is evident from the transmembrane potential profiles at the hyperpolarized (negative) and depolarized (positive) poles(Fig. 7). that both amplitude and sharpness increase significantly when the electric field rises from $1~\mathrm {kV/cm}$ to $4~\mathrm {kV/cm}$. Peaks typically occur at around $t=2~\upmu \textrm{s}$, corresponding to maximum membrane polarization regions most likely to experience electroporation or pore formation. The subsequent voltage decline reflects membrane relaxation or resealing. These findings confirm that electric field strength directly influences membrane permeability dynamics in CTCs.

**Fig. 9:**
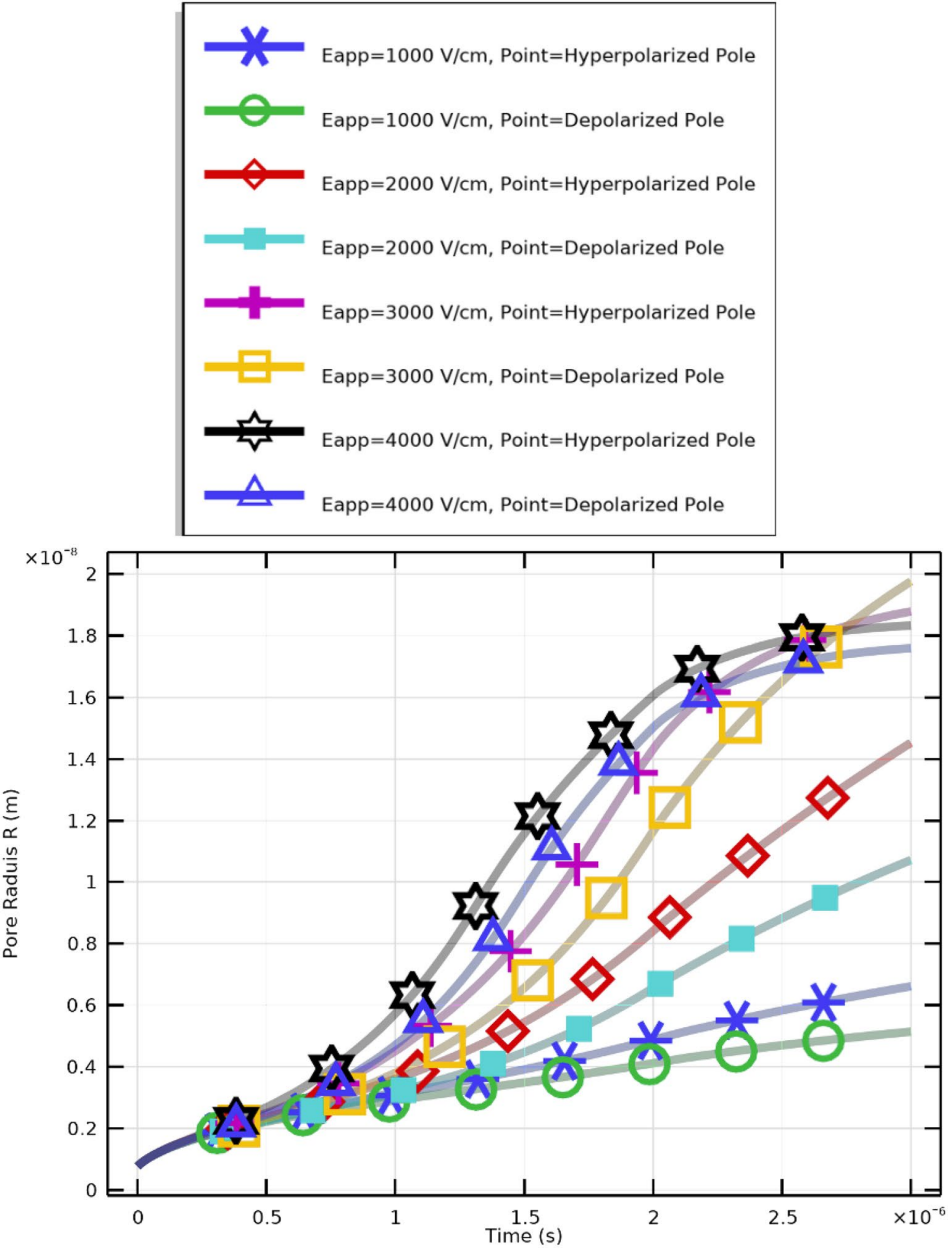
Pore radius variation with time under $2~\upmu \textrm{s}$ excitation and electric field strengths of 1–4 kV/cm.

### Pore formation and membrane resealing

Figure 9 illustrates the temporal evolution of pore radius (*R*) under different applied electric field strengths ($E_{\text {app}}$). Distinct colors represent variations between hyperpolarized (negative transmembrane potential) and depolarized (positive transmembrane potential) poles. The curves show that pore size grows faster and to larger values as electric field strength increases, consistent with physical predictions that stronger electric fields accelerate pore nucleation and expansion.

Critical pore formation can therefore be achieved even at lower electric field strengths by aligning pulse duration with cellular charging time. However, higher field strengths yield greater pore density and increased transmembrane potential. Effective membrane resealing is observed for pulses of $2~\upmu \textrm{s}$ with $E_{\text {app}} \ge 2~\mathrm {kV/cm}$, whereas pores remain limited for weaker fields. An electric field of approximately $3~\mathrm {kV/cm}$ achieves maximum pore density. Both pulse duration and field strength are crucial for regulating membrane permeability and resealing in CTCs.

**Fig. 10:**
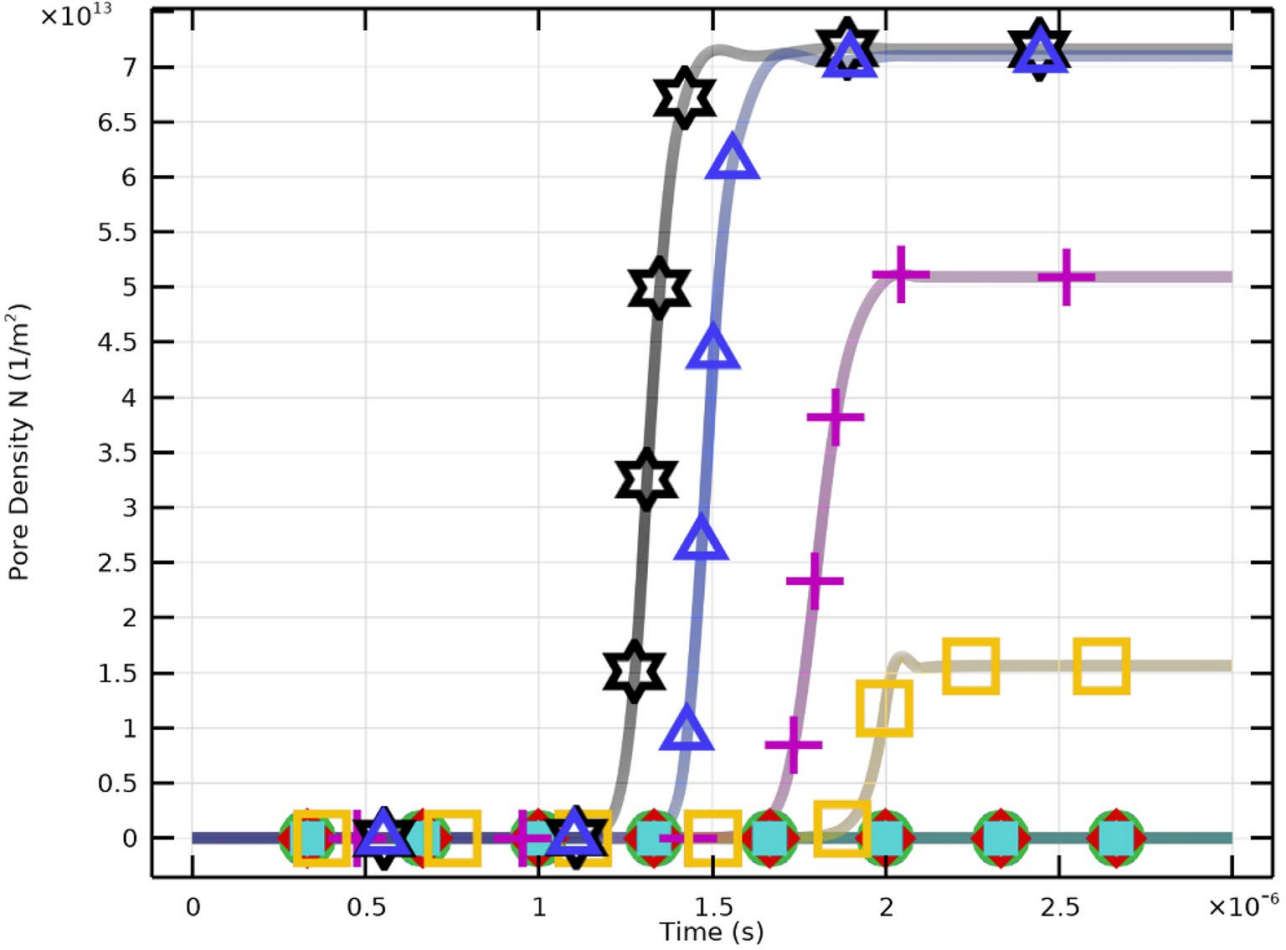
Pore density and membrane resealing under excitation conditions of $2~\upmu \textrm{s}$ at various electric field strengths.

**Fig. 11:**
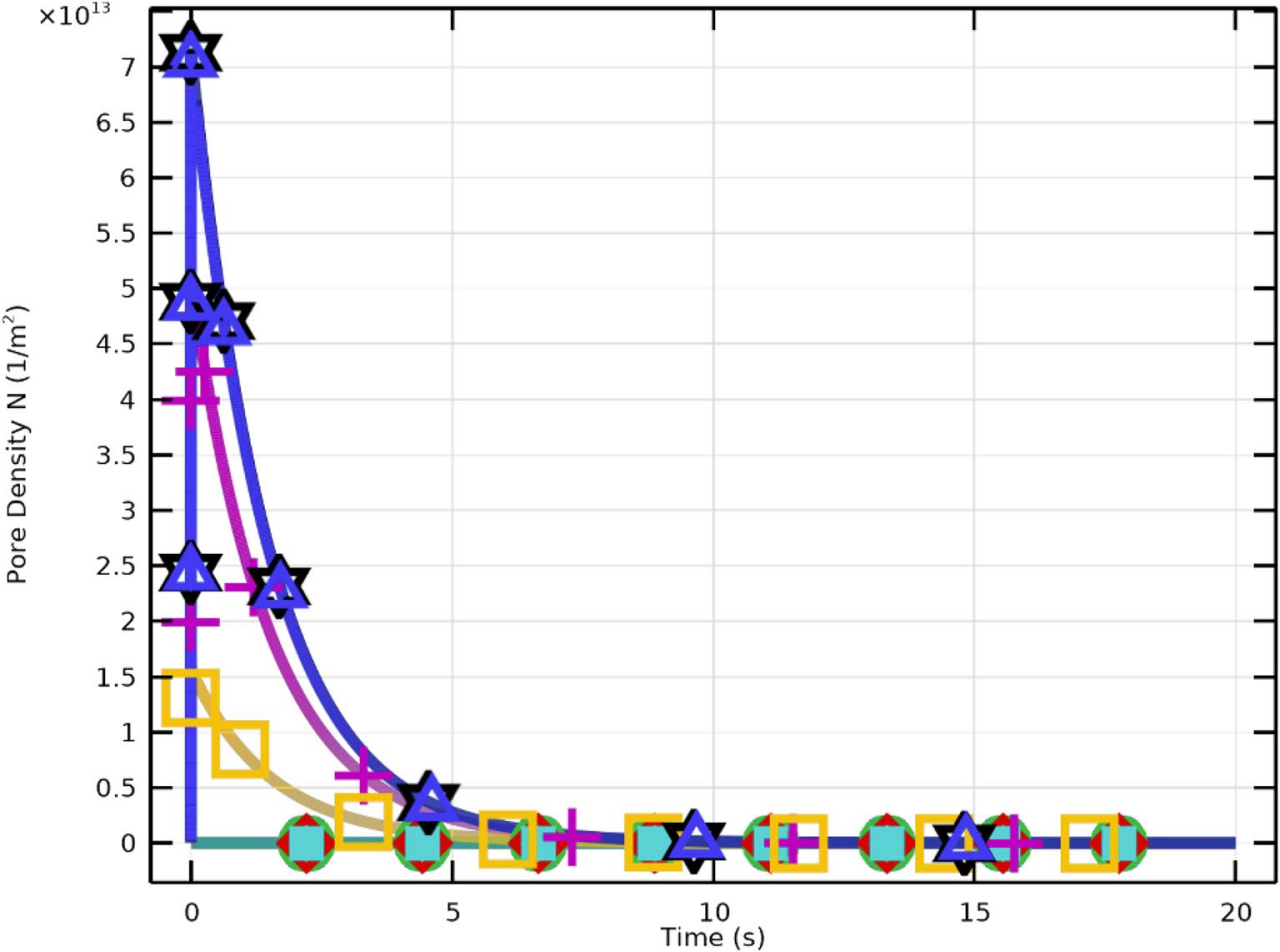
Electric field strength (1–4 kV/cm), pore density, and membrane resealing with $2~\mu \textrm{s}$ excitation.

Figure 10 depicts the temporal dynamics of pore density (*N*) and membrane resealing. Higher field strengths (e.g., 3–4 kV/cm) result in rapid pore formation followed by a saturation plateau indicating the limiting pore density. Figure 11 shows that pore density drops drastically following the pulse, reflecting effective resealing. Lower fields produce less pore density and slower resealing.

Thus, optimized electroporation protocols should employ field strengths and durations that enable rapid pore formation followed by fast resealing, ensuring reversible electroporation with minimal damage.

**Fig. 12:**
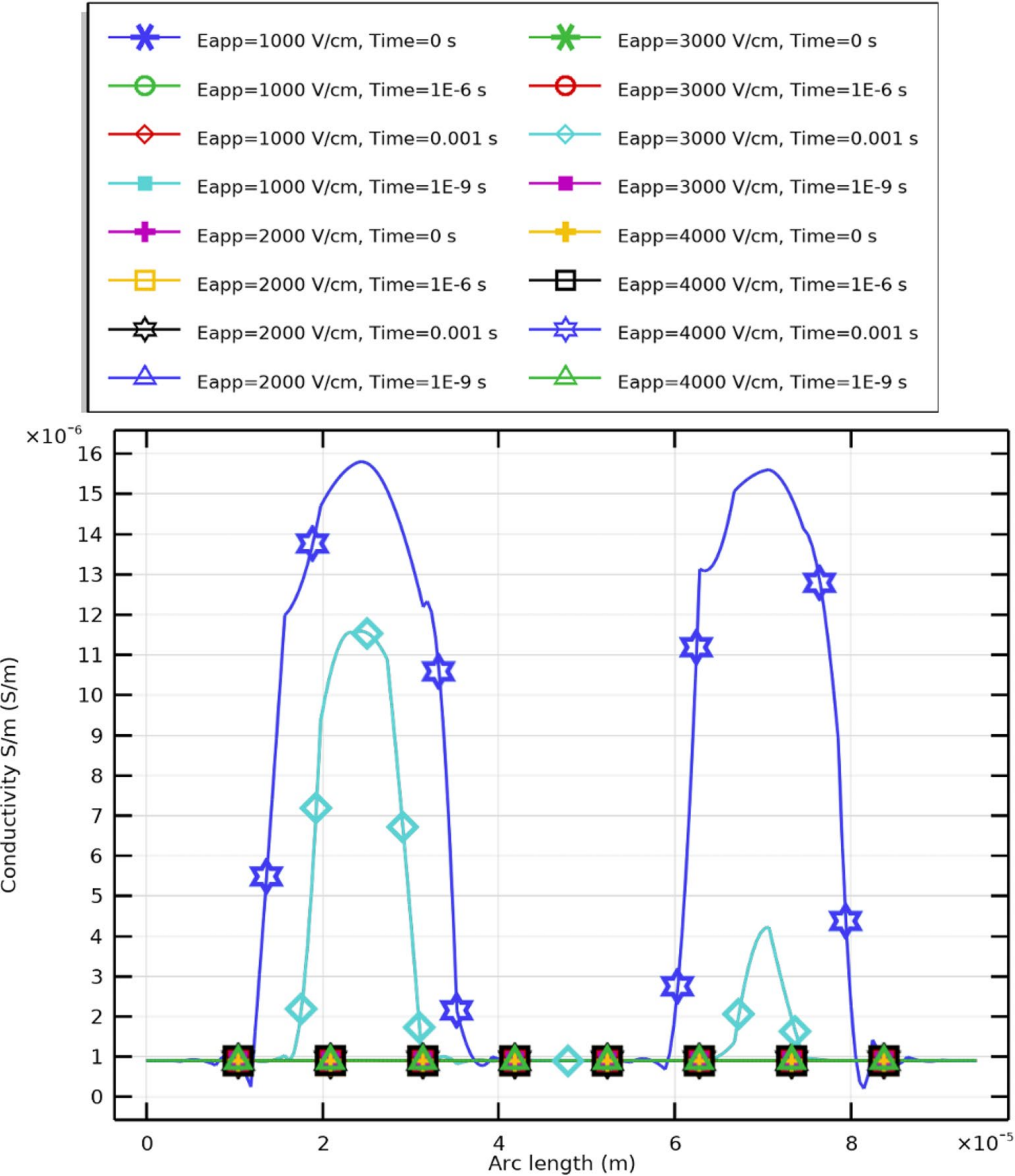
Membrane conductivity variation with $2~\mu \textrm{s}$ excitation and electric fields between 1 and 4 kV/cm.

As shown in Fig. 12, membrane conductivity increases sharply under strong fields, peaking at $9.7\times 10^{-5}~\mathrm {S/m}$ (for $E_{\text {app}}=4000~\mathrm {V/cm}$ at $t=2~\mu \textrm{s}$), before decreasing to approximately $9.3\times 10^{-7}~\mathrm {S/m}$ over the next second. The increase in conductivity directly correlates with enhanced pore formation, consistent with the predictions of the RC circuit model of the cell. As the transmembrane potential decays post-pulse, conductivity decreases, reflecting gradual membrane resealing.

### Localized field effects and pore distribution

The contour plots (Figs. S1, S2) visualize the electric displacement field norm ($\mathrm {C/m^2}$) at 20 s following a $2~\upmu \textrm{s}$ pulse. These illustrate polarization effects across the cell membrane under various field strengths.

At $1000~\mathrm {V/cm}$ (Fig. S1), electric displacement peaks near the membrane ($\approx 14.8\times 10^{-20}~\mathrm {C/m^2}$), highlighting strong polarization near the cell boundary. The upper pole exhibits depolarization, while the bottom pole shows distinct hyperpolarization.

Increasing the field to $2000~\mathrm {V/cm}$ (Fig. S2) enhances both the magnitude and gradient of the displacement field. Stronger field localization occurs near the hyperpolarized pole, producing sharper gradients across the membrane and higher charge separation.

At $3000~\mathrm {V/cm}$ (Fig. S3), field intensity further increases, reaching approximately $4.33\times 10^{-11}~\mathrm {C/m^2}$. The displacement becomes more concentrated near the bottom pole, with steeper gradients across the membrane, emphasizing stronger polarization and enhanced electroporation potential.

At $4000~\mathrm {V/cm}$ (Fig. S4), the displacement magnitude reaches up to $4.7\times 10^{-11}~\mathrm {C/m^2}$ and is symmetrically distributed around the membrane. The localized poles merge into a continuous ring of high polarization, indicating uniform membrane activation. Stronger fields reinforce charge separation, broaden the polarization zone, and accentuate the full-membrane electroporation dynamics observed in CTCs.

## The appropriate condition for PLT cells

The investigation was extended to platelet (PLT) cells using the dielectric characteristics listed in Table 1 to analyze their electroporation dynamics. The same mathematical model used for circulating tumor cells (CTCs) was applied here, maintaining identical electrode spacing and dimensions. To perform a comprehensive evaluation, the applied electric field strength ($E_{\text {app}}$) was varied across 10, 20, 30, and 40 kV/cm while the pulse duration was kept constant at $2~\upmu \textrm{s}$. This configuration allows precise control over electroporation behavior, including pore nucleation and resealing dynamics, thereby ensuring optimal membrane permeability while maintaining cellular viability.

**Fig. 13:**
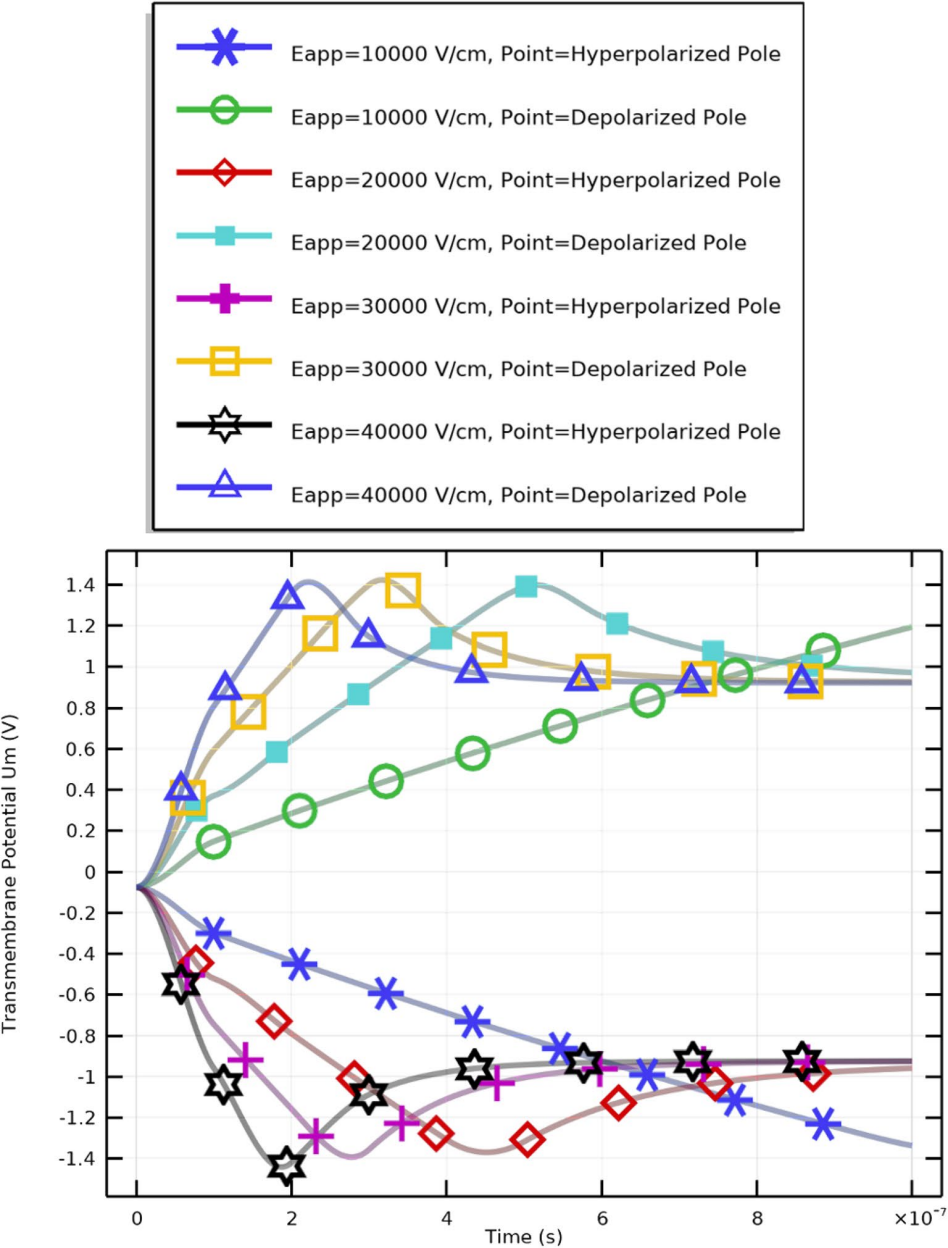
Transmembrane potential at the key selective poles of PLT cells under electric fields of 10–40 kV/cm. The results indicate distinct polarization behaviors at the hyperpolarized and depolarized poles during pulse excitation. The field must be sufficiently strong to induce effective electroporation; intensities between 10 and 40 kV/cm achieve the desired membrane permeabilization.

**Fig. 14:**
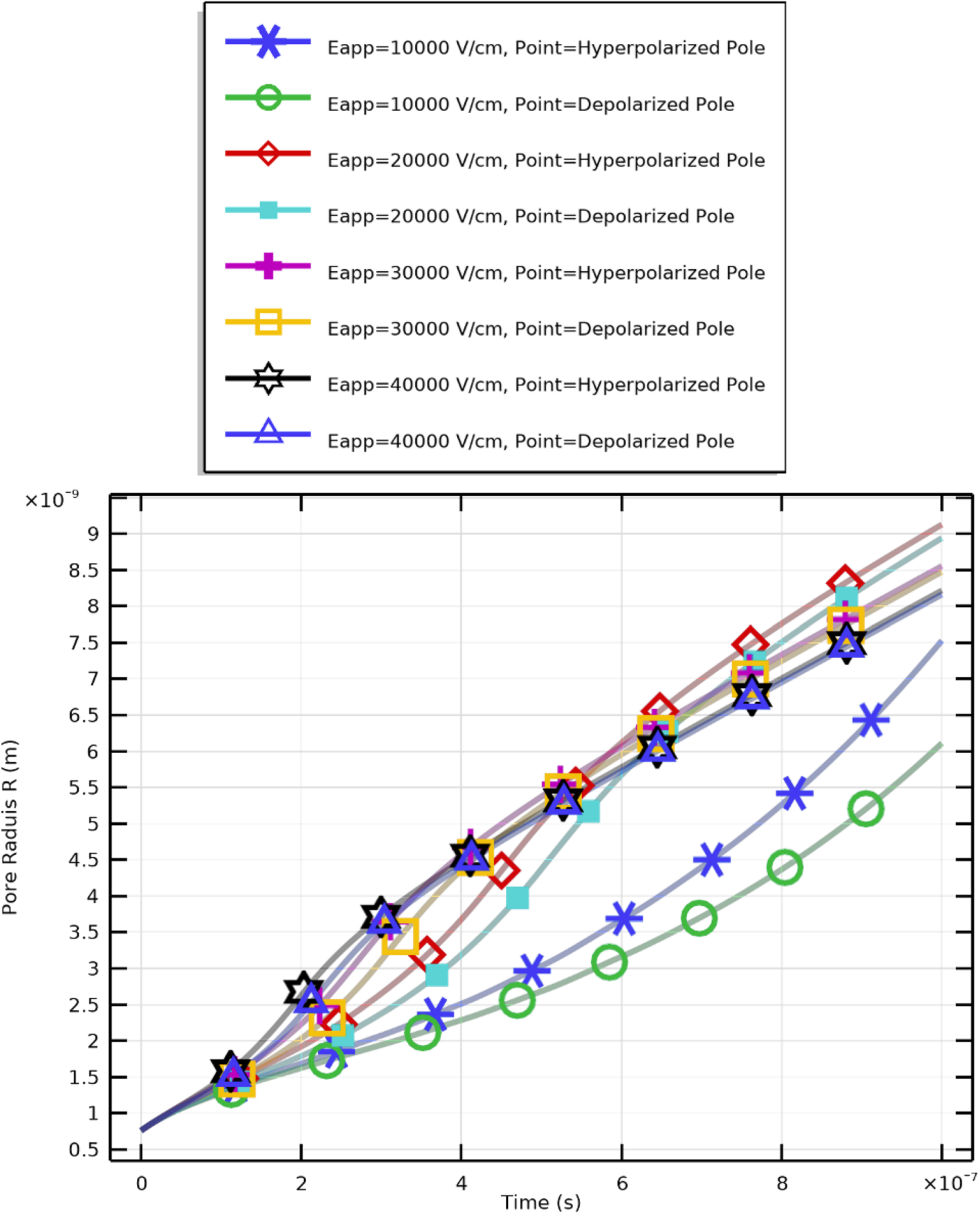
Temporal evolution of the pore radius (*R*) in PLT cells under a $2~\upmu \textrm{s}$ excitation and applied field strength $E_{\text {app}}=[10{:}10{:}40]~\mathrm {kV/cm}$. The maximum pore radius occurs at $20~\mathrm {kV/cm}$: $R_{\text {max}}=9\times 10^{-9}~\textrm{m}$ (hyperpolarized pole) and $8\times 10^{-9}~\textrm{m}$ (depolarized pole).

Figure 13 and subsequent plots illustrate the mathematical analyses for PLT cells under different applied field strengths. Figure 13 shows the evolution of the transmembrane potential ($V_\textrm{m}$) at both the hyperpolarized (negative) and depolarized (positive) poles, while Fig. 14 presents the temporal evolution of the pore radius (*R*). Each symbol and color in the plots represent distinct combinations of membrane pole and electric field intensity, as described in the Supplementary Materials.

**Fig. 15:**
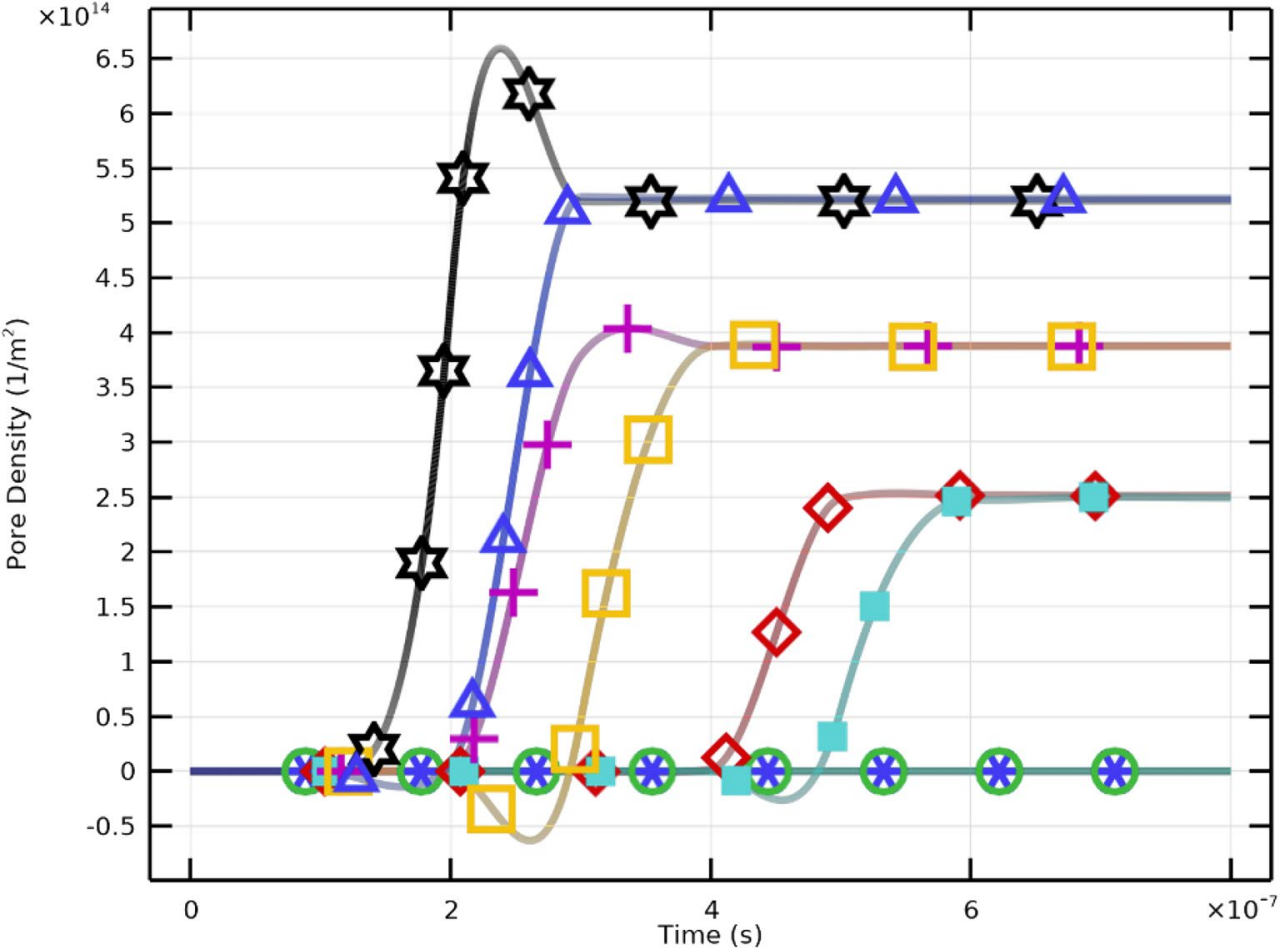
Pore density evolution and membrane resealing dynamics in PLT cells under $2~\mu \textrm{s}$ pulses.

**Fig. 16:**
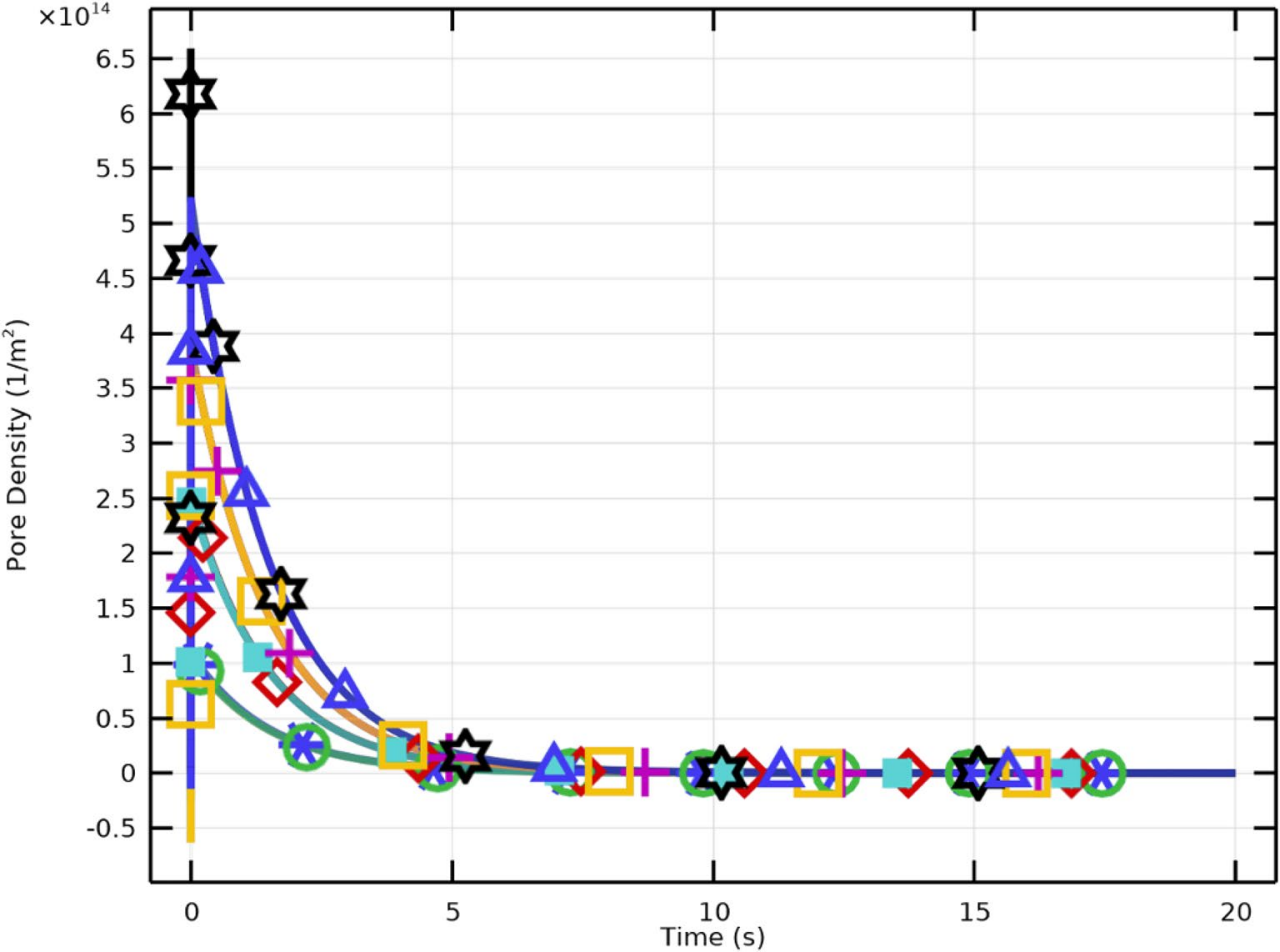
Membrane resealing kinetics in PLT cells after pulsed electric field excitation of $2~\mu \textrm{s}$.

The results demonstrate that stronger electric fields accelerate pore nucleation and expansion in PLT membranes. Higher applied voltages (30–40 kV/cm) cause faster and more pronounced increases in pore radius, confirming the nonlinear dependence between $E_{\text {app}}$ and membrane permeabilization. Importantly, when the excitation pulse duration matches the membrane charging time, critical pore formation can still occur at lower field intensities (10–20 kV/cm). However, higher fields yield elevated pore density (Fig. 15) and enhanced $V_\textrm{m}$, while moderate fields ($E_{\text {app}}\!\ge \!20~\mathrm {kV/cm}$) promote effective resealing (Fig. 16). The maximum pore density corresponds to $E_{\text {app}}=30~\mathrm {kV/cm}$, establishing it as the optimal electroporation parameter for PLTs.

**Fig. 17:**
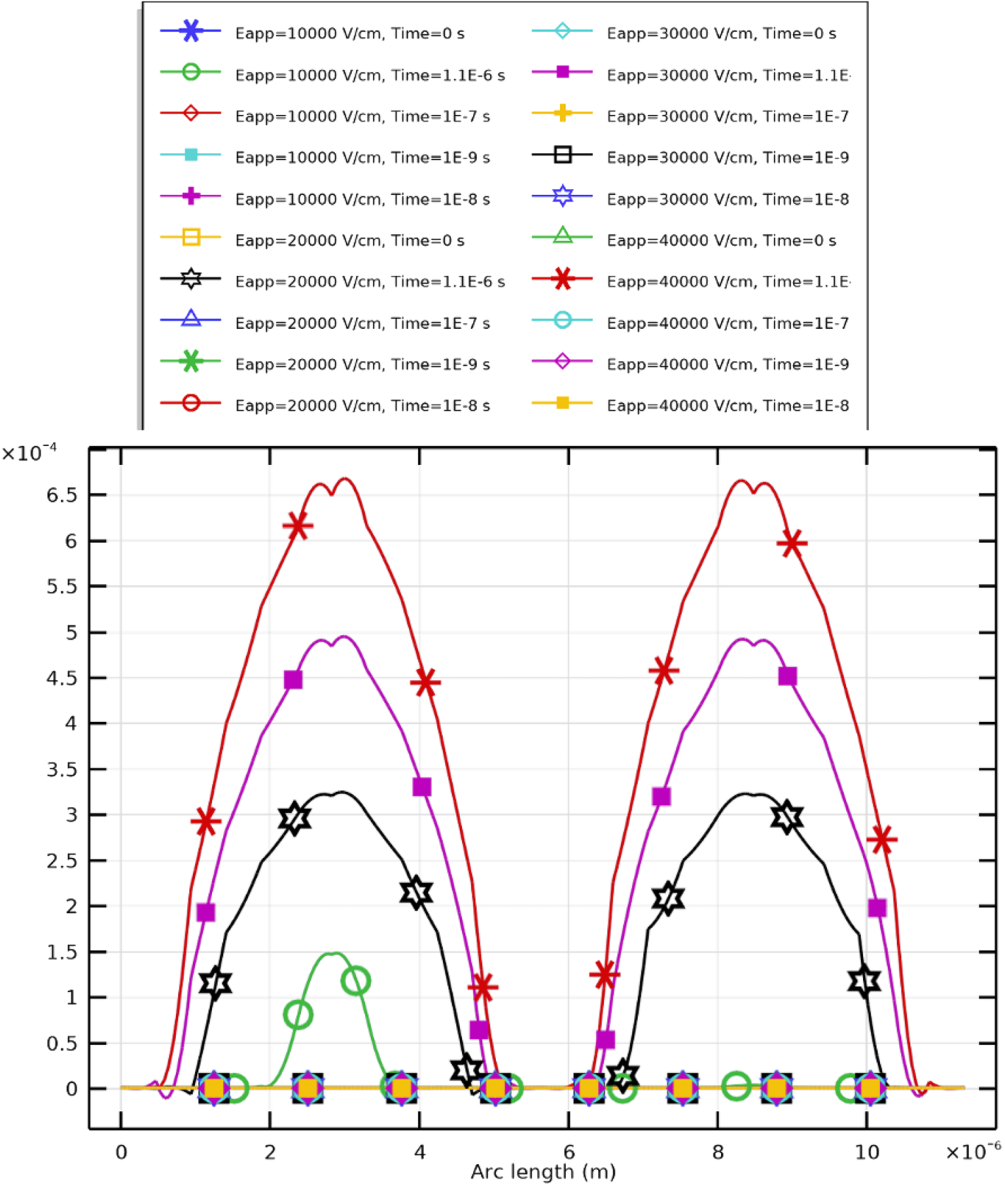
Variation of membrane conductivity in PLT cells under pulsed excitation ($2~\mu \textrm{s}$, $E_{\text {app}}=[10{:}10{:}40]~\mathrm {kV/cm}$). The peak conductivity occurs near $1~\upmu \textrm{s}$, coinciding with maximum pore formation, followed by a gradual decline due to resealing.

Collectively, these results highlight the crucial influence of electric field intensity and pulse duration on both pore evolution and membrane conductivity (Fig. 17), confirming that an electric field range of 10–40 kV/cm provides efficient and safe electroporation conditions for PLTs. This aligns with experimental findings that smaller cells such as PLTs require higher field strengths due to their reduced size and distinct membrane composition ^52^. Maintaining short pulses ($2~\upmu \textrm{s}$) minimizes undesirable thermal effects such as Joule heating, preserving cell integrity and functionality.

### Transmembrane potential dynamics

Three stages can be identified in the transmembrane potential profile shown in Figure 13:

A. Region A: Charging phase ($T = 1.365\times 10^{-7}~\textrm{s}$).
B. Region B: Pore nucleation ($5.01\times 10^{-7}~\textrm{s}> T > 1.365\times 10^{-7}~\textrm{s}$).
C. Region C: Pore evolution ($T > 5.01\times 10^{-7}~\textrm{s}$).

The charging phase begins immediately after pulse application and ends with the first pore formation. Once $V_\textrm{m}$ exceeds approximately $\pm 1~\textrm{V}$, pores nucleate rapidly, followed by a slower pore evolution phase. As membrane permeability rises, the charging time correspondingly decreases. Factors such as buffer composition and membrane cholesterol content modulate the efficiency of electroporation by altering conductivity and pore kinetics. These conditions determine the threshold and extent of electrophysical membrane disruption necessary for controlled, reversible electroporation of PLTs.

## Comparison between PLT and CTC behavior

Comparing the three electroporation stages charging, pore nucleation, and pore evolution in CTCs and PLTs reveals clear distinctions primarily influenced by cell radius. The smaller radius of PLTs leads to a faster establishment of $V_\textrm{m}$ across the membrane due to higher capacitance per unit volume, resulting in much shorter characteristic times for all three regions. Consequently, PLTs respond more rapidly to electric pulses than CTCs, experiencing earlier onset of nucleation and evolution phases. This confirms that smaller cells charge faster and exhibit faster electroporation kinetics, reinforcing the strong dependence of electroporation time constants on cell geometry.

As shown in Fig. 14, higher field amplitudes enhance pore generation and cause temporal plateaus in pore radius beyond 20 kV/cm, implying saturation. The initial growth phase corresponds to pore nucleation, followed by an expansion phase and subsequent stabilization, even under intensified fields. A balance therefore exists: higher fields generate more pores but not necessarily larger ones. Optimal pore formation occurs at moderate intensities, ensuring both efficient molecular transport and maintained cellular viability.

### Pore formation and membrane resealing

Figures 15 and 16 illustrate the dynamic relationship between electric field intensity, pore density, and resealing. Pore density rises sharply following pulse initiation and reaches its maximum at approximately $40~\mathrm {kV/cm}$, aligned with the increase in pore size at high fields. Subsequently, rapid pore closure indicates efficient resealing, confirming that the selected 2 $\upmu$s pulse ensures reversible permeabilization. These trends emphasize the importance of optimizing both field amplitude and pulse duration to achieve effective, transient membrane permeability while preserving overall integrity.

During electroporation, PLT membrane conductivity peaks near $t\approx 1~\upmu \textrm{s}$, then slightly declines at the end of the $2~\upmu \textrm{s}$ excitation, consistent with membrane recovery. These variations reflect localized changes in ion transport associated with pore formation. PLTs exhibit reversible electroporation, allowing them to retain functionality after treatment. The transient conductivity is governed by ion fluxes (mainly $\mathrm {Na^+}$, $\mathrm {K^+}$, and $\mathrm {Ca^{2+}}$) and highlights the delicate balance between efficient electroporation and cell viability.

### Localized field effects and pore distribution

Fig:S5 maps the electric displacement field (*D*) across the PLT membrane at an applied field of 10 kV/cm. Significant polarization appears at the hyperpolarized pole with a maximum $D\!\approx \!5.75\times 10^{-11}~\mathrm {C/m^2}$. This asymmetry reflects differential polarization governed by intrinsic cellular geometry and dielectric properties. The applied pulse induces local membrane deformation and nanopore formation, advancing electroporation. As the field strength increases, the displacement field intensifies and spreads uniformly across the membrane surface, ensuring homogeneous permeabilization and consistent molecular transport.

Supplementary Figs. S6–S8 show the progression of membrane polarization as the electric field increases to 20, 30, and 40 kV/cm, respectively. The contour plots reveal that increasing $E_{\text {app}}$ strengthens the displacement field and expands its spatial distribution. At 20 kV/cm, the field is mainly confined near the poles; at 30 kV/cm, it becomes more uniform; and at 40 kV/cm, it fully envelops the membrane. This evolution matches theoretical electrodynamic predictions, where field saturation leads to complete membrane activation. These patterns demonstrate the robustness of the simulation in capturing field-induced cellular responses, consistent with behaviors observed experimentally in CTCs under similar operating conditions.

Together, these results validate that increasing the applied electric field both amplifies and broadens the displacement field across the membrane, ensuring predictable and effective electroporation. The PLT modeling outcomes offer a quantitative benchmark for comparing membrane electrodynamics across cell types and provide clear guidance for optimizing pulse parameters in biomedical and therapeutic applications.

## The appropriate condition for WBC cells

White blood cells (WBCs) are essential components of the immune system, responsible for defending the body against infections and foreign agents. Constantly circulating through the bloodstream and tissues, these cells coordinate complex immune responses to maintain homeostasis and overall health. Understanding how WBCs behave under the different conditions comparable to those of circulating tumor cells (CTCs) is vital for unveiling their distinct biophysical behaviors.

In this study, the applied electric field strength ($E_{\text {app}}$) for WBCs was precisely controlled and varied at 1, 2, 3, and $4~\mathrm {kV/cm}$, mirroring the field range previously used in CTC analyses. This controlled parameter variation enables direct comparison of cellular responses, elucidating differences in electroporation thresholds, membrane permeabilization, and recovery behaviors between WBCs and CTCs.

### Transmembrane potential dynamics

**Fig. 18:**
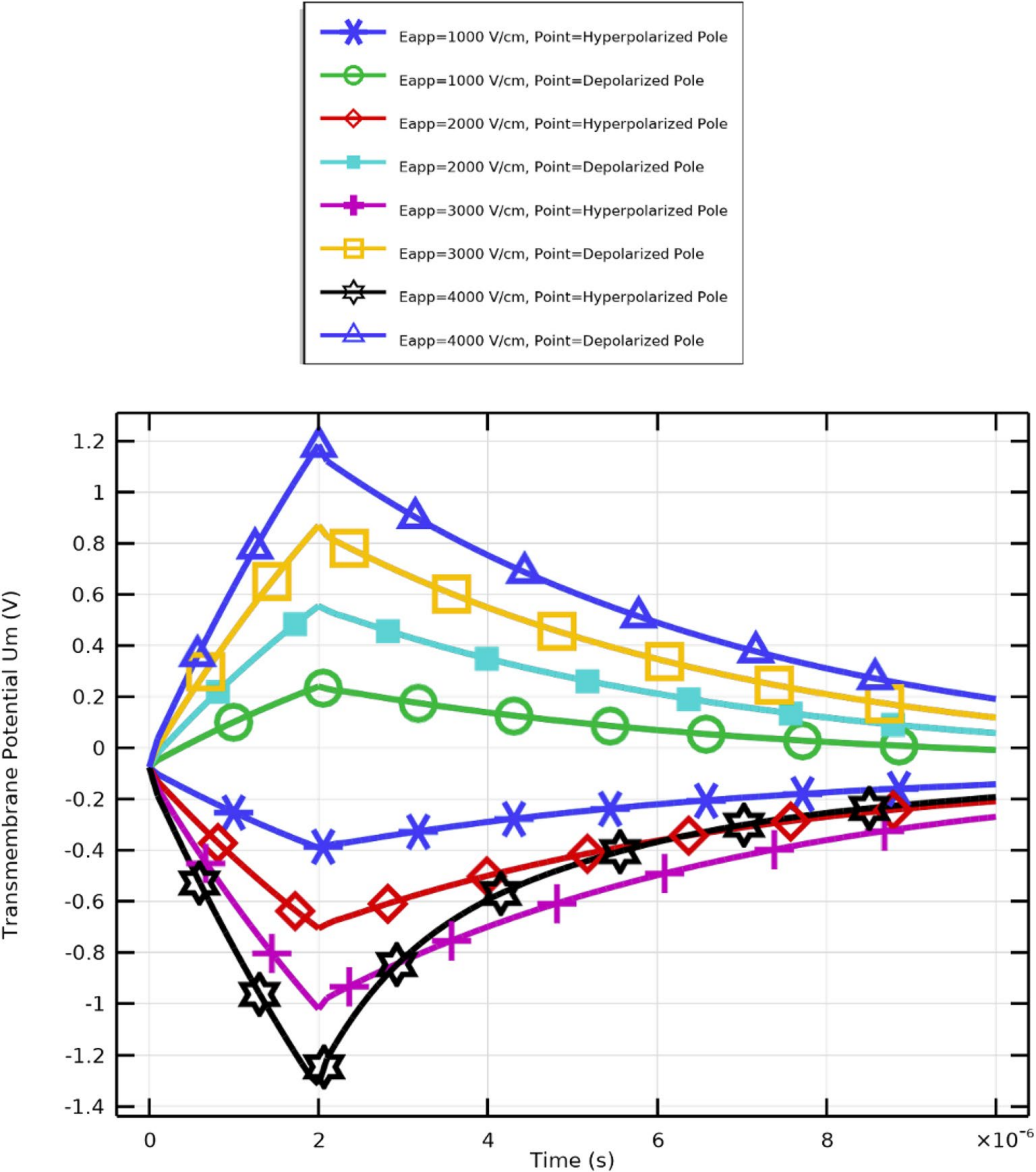
Time evolution of transmembrane potential ($V_\textrm{m}$) in WBC membranes under pulsed electric field excitation.

When subjected to a pulsed electric field, WBC membranes exhibit a transient polarization response in their transmembrane potential ($V_\textrm{m}$). The rapid rise and gradual decay of $V_\textrm{m}$ over time are illustrated in Fig. 18. Upon field application, the hyperpolarized pole attains peak potentials of approximately $+1.1~\textrm{V}$ at $t = 2~\upmu \textrm{s}$, while the depolarized pole simultaneously reaches about $-1.2~\textrm{V}$. This asymmetric response arises from cell geometry, membrane resistance, capacitance, and the nonuniform local field distribution.

The observed potential thresholds correspond closely to the onset of electroporation, during which nanopores form across the lipid membrane. Due to their larger diameter and distinct membrane composition, WBCs exhibit slower charging and relaxation kinetics compared with platelet (PLT) cells. PLTs, being smaller and possessing a higher surface-to-volume ratio, reach peak potentials faster and reseal sooner upon pulse termination. These findings underscore the importance of tailoring electroporation parameters specifically, field strength and pulse duration to the physical and electrical characteristics of each cell type to maintain both permeabilization efficacy and cell viability.

### Pore formation and membrane resealing

**Fig. 19:**
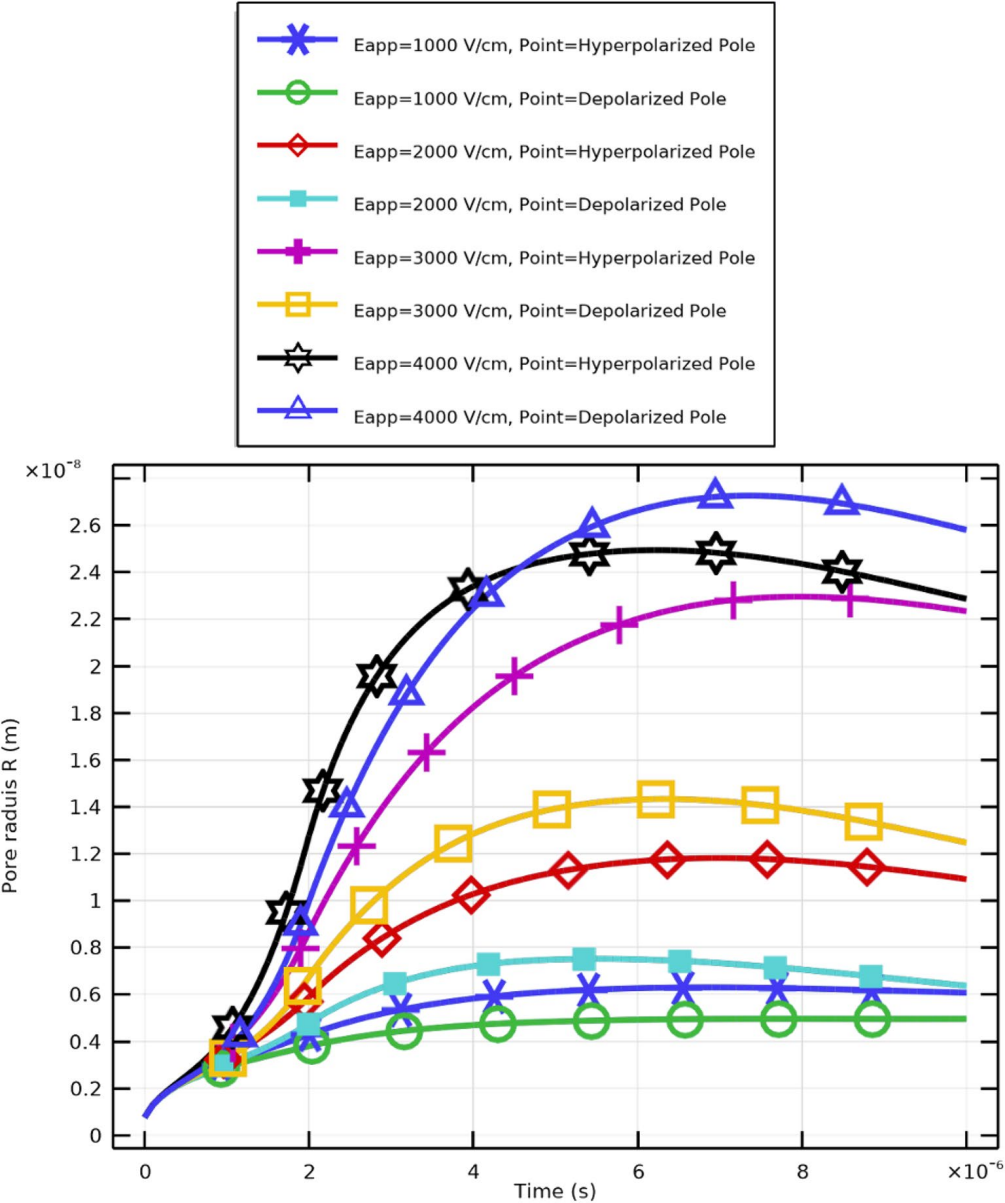
Pore radius dynamics and membrane resealing in WBC membranes subjected to pulsed electric fields.

**Fig. 20:**
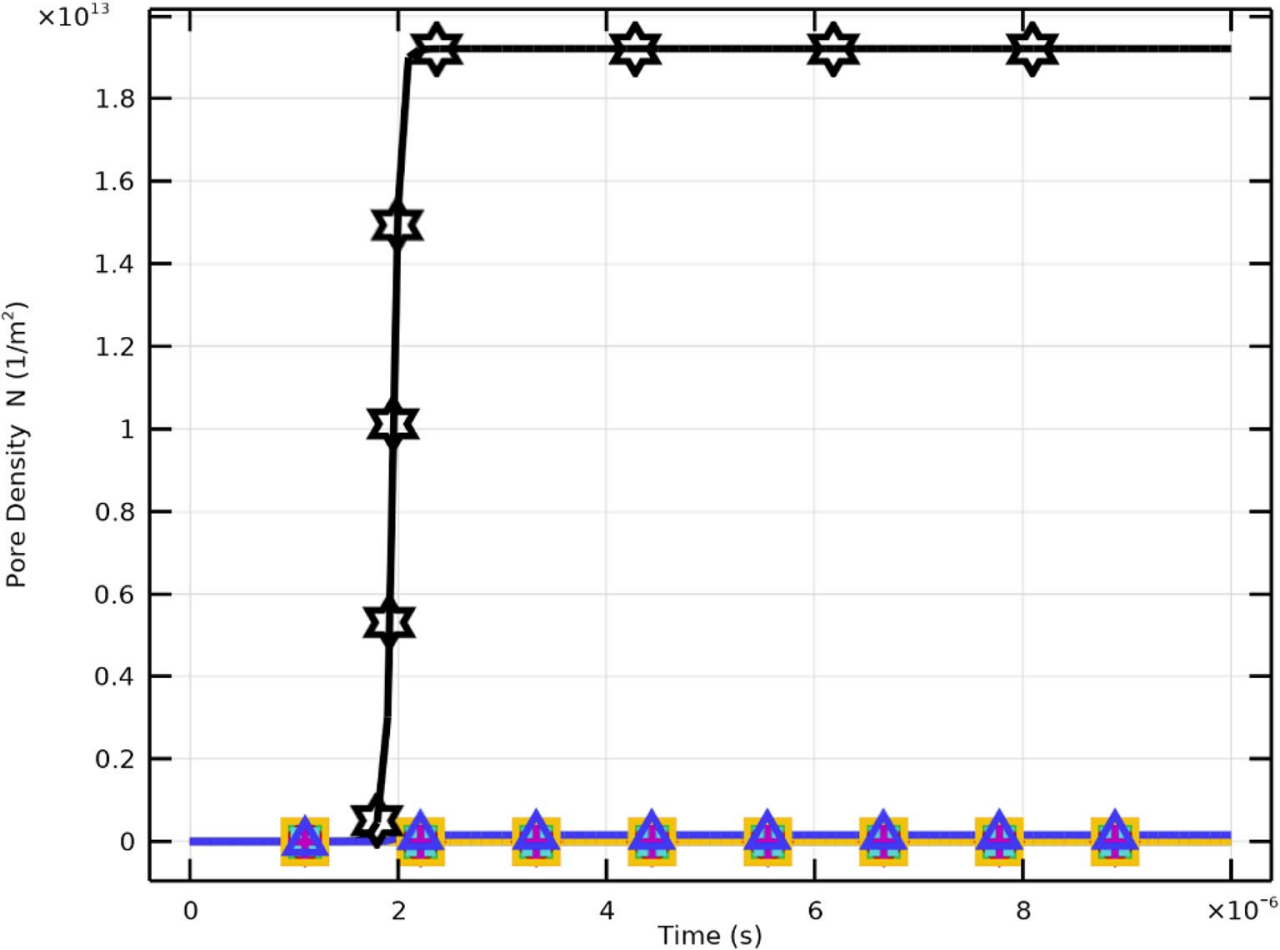
Time evolution of pore density in WBC membranes under electric pulses of $2~\upmu \textrm{s}$ at various field intensities.

**Fig. 21:**
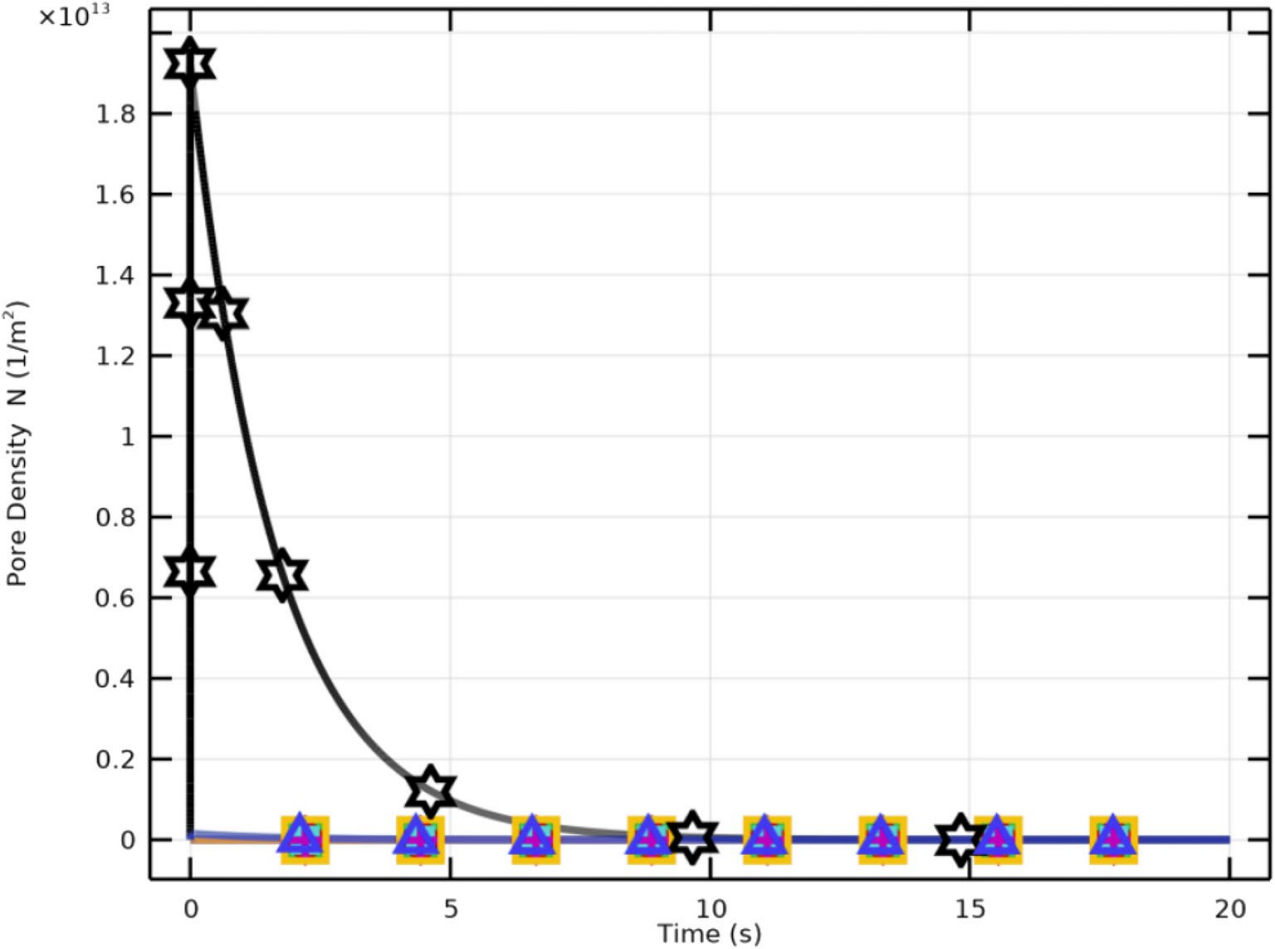
Membrane resealing behavior following pulsed electric field excitation of WBC cells.

The dynamics of pore radius and density under various field strengths are shown in Figs. 19, 20 and 21. Increasing $E_{\text {app}}$ leads to a field-dependent rise in membrane permeabilization. At $E_{\text {app}}=4~\mathrm {kV/cm}$, the depolarized pole, the largest mean pore radius is defined, while the hyperpolarized pole reaches a slightly smaller peak value. Despite this, the pore density is typically higher at the hyperpolarized pole (Supplementary Figs. S10, S11 ), indicating localized enhancement of the electric field at this site.

At the hyperpolarized pole, maximum pore density ($1.9183\times 10^{13}~\mathrm {m^{-2}}$) occurs approximately $2.2~\upmu \textrm{s}$ after excitation. The rapid formation of pores immediately after the pulse, followed by membrane resealing, confirms efficient and reversible electroporation. These results highlight the interplay between electric field intensity, membrane polarity, and temporal evolution critical factors for controlled molecular transport in WBCs.

### Localized field effects and pore distribution

The strong asymmetry in pore density between the hyperpolarized (HP) and other membrane regions (Figs. 20, 21) arises from intrinsic biophysical and electrochemical phenomena. Cellular geometry amplifies local field strength at the HP region, enhancing charge accumulation and polarization. Once the local $V_\textrm{m}$ surpasses $\pm 1~\textrm{V}$, rapid pore nucleation occurs, leading to high-density permeabilization zones. Conversely, membrane sectors exposed to weaker or misaligned fields exhibit limited electroporation, resulting in heterogeneous pore formation across the cell surface.

This spatially nonuniform behavior demonstrates that electroporation in WBC membranes is inherently localized. Controlled manipulation of this effect especially targeting regions near the HP pole enables selective and efficient molecular delivery without compromising global membrane integrity.

### Membrane conductivity dynamics

**Fig. 22:**
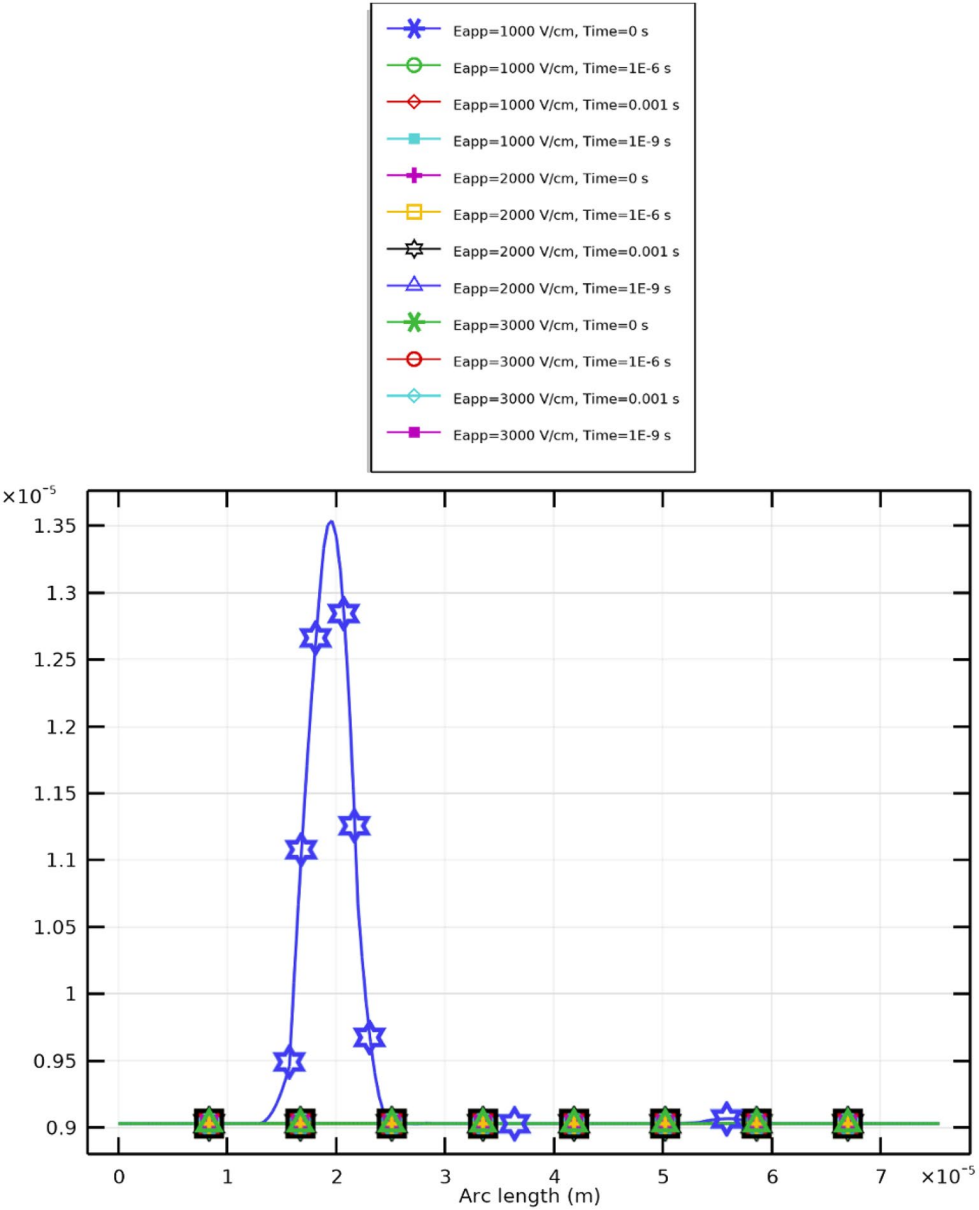
Temporal evolution of membrane conductivity in WBC cells under a $2~\upmu \textrm{s}$ excitation and electric field strength of $E_{\text {app}}=[1{:}1{:}4]~\mathrm {kV/cm}.$.

The evolution of membrane conductivity under pulsed excitation is shown in Fig. 22. WBCs exhibit substantial spatial heterogeneity, with conductivity at the HP pole significantly exceeding other regions. This increase reflects enhanced ion transport through transient aqueous pores and a concomitant drop in local resistance. Conversely, regions exposed to weaker fields preserve baseline conductivity. Such differentiation underscores the importance of electric field distribution and cell orientation in governing electroporation outcomes.

**Fig. 23:**
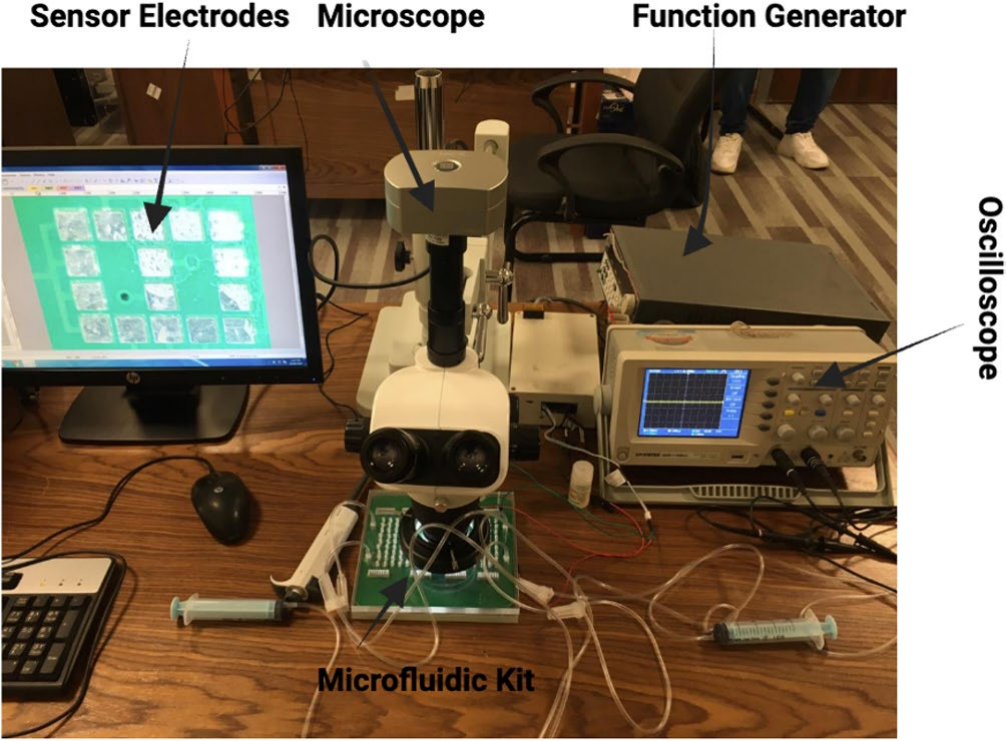
Experimental setup used to characterize cellular response as a function of impedance during electroporation. THP-1 cells are loaded into a microfluidic device integrated with sensor electrodes and positioned under an optical microscope for real-time visualization. An external function generator applies controlled electrical pulses to induce electroporation, while the resulting impedance changes are monitored using an oscilloscope. This configuration enables simultaneous optical observation and electrical measurement to quantify cell membrane response under electroporation conditions.

**Fig. 24:**
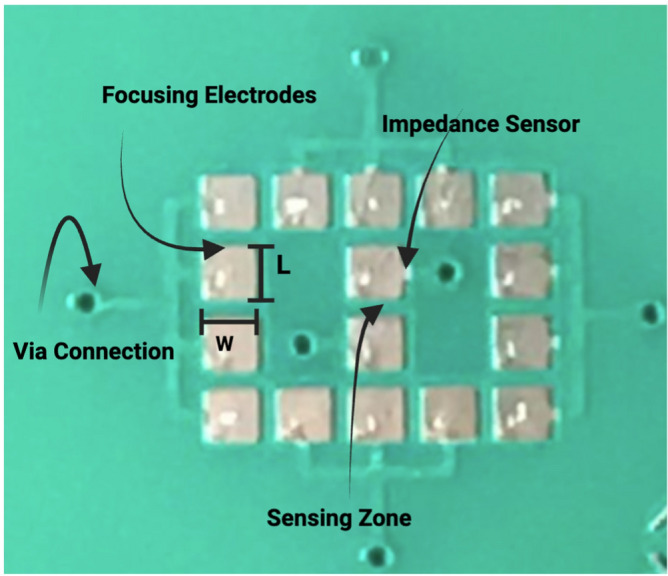
Optical micrograph of the microfabricated electrode array integrated within the microfluidic device. The central impedance sensor defines the sensing zone where cell impedance is measured during electroporation. Surrounding focusing electrodes are used to localize and control the electric field distribution. Electrical routing is achieved through via connections, enabling independent excitation and measurement of the electrodes for precise impedance-based characterization of cellular response. Electrode lateral dimensions: $L = w = 600$ µm; inter-electrode spacing: 200 µm.

**Fig. 25:**
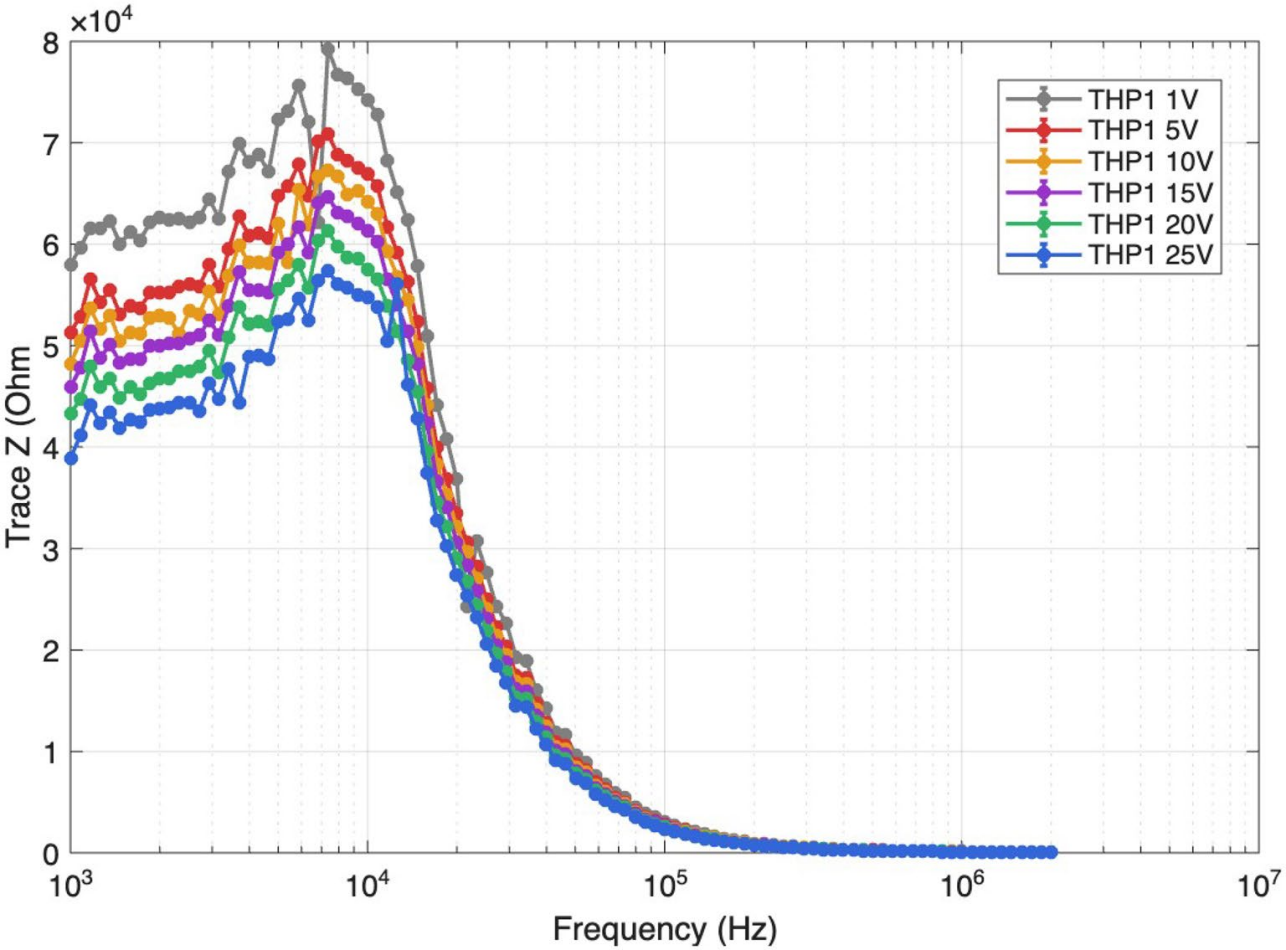
Voltage-dependent impedance magnitude spectra of THP-1 cells. Bode plots of $|Z|$ measured from $10^{3}$ to $2 \times 10^{6}$ Hz under baseline conditions (1 V) and after electroporation at 5–25 V. A progressive decrease in impedance with increasing voltage is observed across the frequency range, most prominently at low frequencies, indicating enhanced conductivity following membrane permeabilization.

Targeted field application can therefore optimize conductivity and transport at specific loci while minimizing nonspecific membrane damage an essential consideration for therapeutic and diagnostic applications employing pulsed-electric-field technologies.

As demonstrated in Supplementary Figs. S9–S12 , electric displacement field distributions (${\bf D}$) in WBC membranes depend strongly on applied field intensity. At lower fields (1–$2~\mathrm {kV/cm}$), displacement is confined near electrode-facing regions, with limited spatial spread and moderate intensity. Increasing $E_{\text {app}}$ to 3–$4~\mathrm {kV/cm}$ expands both field magnitude and distribution over the membrane, achieving homogeneous polarization and enabling nanopore nucleation across larger areas.

This progressive enhancement of the displacement field reflects the fundamental biophysical mechanism of charge accumulation and membrane polarization. At higher intensities, the field penetrates deeper into the cytoplasm and influences intracellular structures, altering ionic transport and organelle behavior. Proper tuning of field parameters is thus essential to balance surface electroporation and intracellular effects, maximizing molecular delivery efficiency while maintaining cell viability in biomedical applications.

## Experimental validation: impedance-based characterization of THP-1 cell electroporation

To complement the computational framework presented in the preceding sections and to provide direct experimental evidence of field-induced membrane permeabilization, a dedicated microfluidic platform integrating impedance sensing with controlled electric field application was developed. This section describes the experimental setup, device architecture, and the impedance spectroscopy measurements performed on THP-1 monocytic cells subjected to increasing electroporation voltages. THP-1 cells were selected as a representative suspended mammalian cell line owing to their well-characterized dielectric properties, spherical morphology in suspension, and relevance as a model for circulating white blood cells in biomedical microfluidic applications. The experimental results are analyzed in terms of three complementary impedance observables magnitude, reactive component, and series resistance to establish a comprehensive electrical signature of the electroporation process and to validate the membrane conductivity trends predicted by the computational model.

### Experimental setup and device architecture

To investigate the relationship between cellular impedance and membrane response during electroporation, a dedicated experimental platform was developed that combines microfluidic confinement, integrated impedance sensing, and real-time optical monitoring Fig. 23. The core of the system is a microfabricated electrode array embedded within a microfluidic channel, comprising a central impedance sensor that defines the active sensing zone and surrounding focusing electrodes designed to localize and shape the electric field distribution within the device Fig. 24. The electrodes possess lateral dimensions of $L = w = 600$ µm and an inter-electrode spacing of 200 µm. Electrical routing is achieved through via connections that allow independent excitation and measurement of individual electrode pairs, thereby enabling precise impedance-based characterization at the single-cell or small-population level.

During operation, THP-1 cells suspended in culture medium are loaded into the microfluidic channel and positioned within the sensing zone defined by the central electrode pair. Controlled electrical pulses, generated by an external function generator, are applied across the 200 µm electrode gap to induce electroporation. The applied electric field is therefore defined as $E = V/d$, where $V$ is the applied voltage and $d = 200$ µm is the inter-electrode spacing, allowing systematic variation of the field intensity from sub-threshold to supra-threshold conditions relative to the electroporation onset predicted by the computational model. The resulting impedance changes are monitored in real time using an oscilloscope connected to the sensing electrodes, while simultaneous optical observation is performed through an inverted microscope to correlate electrical measurements with morphological changes in the cell population. This dual-modality configuration enables quantitative assessment of cell membrane response under precisely controlled electroporation conditions.

### Impedance magnitude response of THP-1 cells under increasing electric field

The impedance magnitude ($|Z|$) of THP-1 cells was evaluated over a frequency range of $10^{3}$–$10^{6}$ Hz under applied voltages ranging from 1 to 25 V across the 200 µm sensing gap. The corresponding applied electric field spans from 50 V/cm at 1 V to 1250 V/cm at 25 V, thereby approaching and exceeding the electroporation threshold predicted by the computational model for cells of comparable diameter. The 1 V condition ($E = 50$ V/cm) was designated as the baseline reference, representing the native electrical response of the cell suspension without significant field-induced membrane modification.

At low frequencies (approximately 1–10 kHz), the impedance magnitude under the baseline condition exhibited the highest values, on the order of $10^{5}$–$10^{6}$ $\Omega$. This behavior reflects the dominant capacitive contribution of intact cellular membranes, which function as insulating barriers to ionic current flow at frequencies where the membrane charging time constant governs the impedance response. As the applied voltage increased from 5 V to 25 V, a consistent and progressive reduction in impedance magnitude was observed across the entire frequency spectrum Figure 25. The decrease became particularly evident in the mid-frequency range (approximately 10–100 kHz), where membrane polarization and interfacial effects most significantly influence the electrical response. In this frequency region, the impedance curves progressively shifted downward with increasing applied voltage, indicating enhanced electrical conduction through the cell population as a consequence of field-induced membrane alteration.

At higher voltages (20–25 V), corresponding to electric fields of 1000–1250 V/cm across the sensing gap, the impedance magnitude decreased substantially relative to the 1 V baseline. This reduction is consistent with increased membrane permeability, enhanced transmembrane ionic transport, and a corresponding increase in the effective conductivity of the cell suspension. Since impedance is inversely related to conductivity ($|Z| \propto 1/\sigma$), the observed decrease in $|Z|$ confirms that the effective conductivity of the system increases monotonically with the applied electric field strength.

The progressive decrease in impedance magnitude with increasing voltage provides direct experimental evidence of field-induced alterations in membrane dielectric properties. As the transmembrane potential increases according to $\Delta V_{m} \propto E \cdot r$, where $r$ is the cell radius, the membrane’s capacitive barrier is progressively compromised, allowing greater current flow through the cellular structure via transient aqueous pores. The lowest impedance values were observed under the 25 V condition, consistent with maximal membrane conductance enhancement and the onset of substantial electroporation within the cell population.

The clear voltage-dependent separation of the impedance curves across the measured frequency spectrum demonstrates that impedance magnitude serves as a sensitive electrical indicator of cellular response to externally applied electric fields. The progressive deviation from the 1 V baseline toward lower impedance values at successively higher voltages confirms that impedance spectroscopy provides a quantitative, label-free tool for monitoring membrane integrity, field-induced conductivity changes, and the progression toward electroporation under controlled conditions within the microfluidic sensing region.

### Reactive impedance response of THP-1 cells under increasing electroporation voltage

The reactive component of impedance ($X_{s}$) was measured from $10^{3}$ to $10^{7}$ Hz for THP-1 cells under baseline conditions (1 V) and after exposure to electroporation pulses at 5 V, 10 V, 15 V, 20 V, and 25 V. The reactive impedance provides complementary information to the magnitude response by isolating the capacitive contribution of the cell membrane from the overall impedance, thereby enabling direct assessment of membrane dielectric integrity as a function of the applied electric field.

Consistent with the magnitude response, the baseline condition (1 V) exhibited strongly negative reactance at low frequencies, reflecting the dominant capacitive behavior of intact cellular membranes and their preserved charge storage functionality (Fig. S13). The large negative $X_{s}$ values observed in the 1–10 kHz range indicate a well-preserved insulating lipid bilayer that substantially impedes alternating current flow through capacitive charging and discharging of the membrane dielectric.

As the applied voltage increased, the magnitude of the negative reactance progressively decreased in a dose-dependent manner. Even at 5 V, a clear reduction in the capacitive response was observed relative to the 1 V baseline, indicating the onset of early membrane perturbation at electric field strengths below the predicted full electroporation threshold. Further increases to 10 V and 15 V produced continued suppression of negative $X_{s}$, demonstrating progressive loss of membrane charge storage capacity consistent with the formation of transient conductive pathways through the lipid bilayer. At 20 V and 25 V, the reactive component approached zero across most of the measured frequency spectrum, indicating substantial attenuation of capacitive membrane behavior and a transition toward resistive, conductivity-dominated electrical transport through the permeabilized cell population.

The voltage-dependent suppression of $X_{s}$ is most pronounced at low frequencies, where the membrane capacitance contributes most strongly to the overall impedance response. At higher frequencies ($\ge 10^{5}$ Hz), all voltage conditions converge toward near-zero reactance values, consistent with direct current penetration through the cytoplasm and surrounding electrolyte regardless of the membrane integrity state. This high-frequency convergence confirms that the observed voltage-dependent differences in $X_{s}$ originate specifically from membrane-level alterations rather than from bulk medium or electrode interface effects.

The clear monotonic ordering of the reactance curves with the 1 V condition exhibiting the most negative $X_{s}$ and successive voltage increments producing progressively less negative values through to 25 V directly parallels the observed decrease in impedance magnitude and provides independent confirmation of dose-dependent membrane permeabilization. Together with the $|Z|$ results, the reactive impedance response further establishes impedance spectroscopy as a sensitive, multi-parameter electrical marker of electroporation-induced membrane alteration in THP-1 cells.

### Series resistance response of THP-1 cells under increasing electroporation voltage

The real component of the series impedance ($R_{s}$) was measured from $10^{3}$ to $10^{7}$ Hz under the baseline condition (1 V) and following electroporation at 5–25 V. The series resistance reflects the resistive contribution of the cell membrane and cytoplasm to the overall impedance and provides a direct indicator of the conductive pathways available for ionic current flow through the cellular structure.

Under the 1 V baseline condition, $R_{s}$ exhibited the largest variability at low and mid frequencies, including apparent negative excursions (Figure S14). These negative values do not represent true negative resistance but arise from the strong capacitive dominance of intact cell membranes at low frequencies, which introduces significant phase shifts between the applied voltage and measured current. When the impedance is decomposed into real and imaginary components under conditions of near-$90^{\circ }$ phase angles, small measurement uncertainties or parasitic contributions can produce apparent negative real parts, a well-recognized artifact in impedance spectroscopy of highly capacitive biological samples.

With increasing applied voltage, the $R_{s}$ magnitude progressively decreased and the frequency-dependent variability diminished substantially. At voltages of 15 V and above, the series resistance remained close to zero across most of the measured frequency spectrum, indicating substantial reduction of the membrane resistive barrier and a pronounced shift toward conductivity-dominated electrical transport. This behavior is consistent with the formation of transmembrane pores that provide low-resistance ionic pathways through the previously insulating lipid bilayer, effectively short-circuiting the membrane resistance.

At higher frequencies ($\ge 10^{5}$ Hz), all voltage conditions converged toward near-zero $R_{s}$ values, consistent with the expected dominance of bulk electrolyte and cytoplasmic conductivity at frequencies where the membrane impedance becomes negligible relative to the series resistance of the surrounding medium. The monotonic suppression of $R_{s}$ with increasing applied voltage provides additional confirmation of dose-dependent membrane permeabilization and complements the impedance magnitude and reactive impedance measurements presented in the preceding subsections.

Taken together, the three impedance observables $|Z|$, $X_{s}$, and $R_{s}$ present a self-consistent electrical signature of progressive membrane permeabilization in THP-1 cells subjected to increasing electric field strength. The convergence of all three parameters toward values characteristic of a conductivity-dominated system at voltages of 20–25 V provides strong experimental evidence that substantial electroporation occurs within this voltage range for the 200 µm electrode geometry employed, corresponding to electric field strengths of 1000–1250 V/cm. These experimental observations are in qualitative agreement with the computational predictions of membrane conductivity enhancement and pore formation dynamics presented in the modeling sections of this study, thereby validating the predictive capability of the multiphysics simulation framework.

**Table 2:** Summary of the investigated electroporation and dielectrophoresis parameters for CTCs, platelets (PLTs), and white blood cells (WBCs) under pulsed electric field excitation.

Cell type	Investigated parameter	Observations / numerical insights
Circulating tumor cells (CTCs)	Electric field strength($E_\text {app}$)	Varied from 1 to 4 kV/cm to assess field–membrane interaction behaviour
	Pulse duration	A 2 $\upmu$s pulse duration was selected to align with the characteristic membrane charging time of CTCs
	Transmembrane potential dynamics	Under fields of 1–4 kV/cm, both amplitude and steepness of potential profiles increased significantly. Maximal polar potentials were observed around $t=2~\upmu$s
	Pore formation and resealing	Pore evolution (radius and density) showed rapid growth and higher density with increased $E_\text {app}$; effective resealing occurred for 2 $\mu$s pulses at $E_\text {app} \ge 2~\text {kV/cm}$, with maximum density around 3 kV/cm
	Membrane conductivity	Exhibited a sharp rise under stronger fields, peaking at $9.7\times 10^{-5}$ S/m for $E_\text {app}=4~\text {kV/cm}$ at $t=2~\upmu$s
	Electric displacement field	Visualized at 1000–4000 V/cm after a 2 $\upmu$s pulse; increased from $1.48\times 10^{-19}$ C/m$^2$ near the membrane (at 1 kV/cm) to $4.7\times 10^{-11}$ C/m$^2$ at 4 kV/cm
Platelet (PLT) cells	Electric field strength ($E_\text {app}$)	Varied across 10, 20, 30, and 40 kV/cm
	Pulse duration	Maintained constant at 2 $\upmu$s.
	Transmembrane potential dynamics	Transmembrane potential variations were assessed at selective poles (10–40 kV/cm), showing field-dependent polarization responses
	Pore radius evolution	Maximum pore radii observed at 20 kV/cm were $9\times 10^{-9}$ m (hyperpolarized pole) and $8\times 10^{-9}$ m (depolarized pole)
	Pore density and resealing	Peak pore density occurred at $E_\text {app}=30~\text {kV/cm}$. Resealing proceeded efficiently after the 2 $\upmu$s excitation
	Membrane conductivity and displacement field	Conductivity peaked around 1 $\upmu$s under 10–40 kV/cm excitation; at 10 kV/cm, maximum electric displacement reached $5.75\times 10^{-11}$ C/m$^2$
White blood cells (WBCs)	Electric field strength ($E_\text {app}$)	Precisely controlled and varied at 1, 2, 3, and 4 kV/cm
	Pulse duration	Fixed at 2 $\upmu$s
	Transmembrane potential dynamics	Hyperpolarized pole reached approximately +1.1 V and depolarized pole about –1.2 V at $t=2~\upmu$s
	Pore radius and density dynamics	At $E_\text {app}=4~\text {kV/cm}$, the depolarized pole exhibited the largest mean pore radius. Maximum pore density $(1.9183\times 10^{13}~\text {m}^{-2})$ occurred near 2.2 $\upmu$s at the hyperpolarized pole
	Membrane conductivity	Temporal variation analyzed for 1–4 kV/cm fields, showing field-dependent conductivity increases
	Electric displacement field	Electric displacement patterns illustrated for 1–4 kV/cm, showing progressive increase in field intensity with higher amplitudes

**Table 3:** Comparative assessment of the present integrated DEP–electroporation study versus representative prior works..

Aspect	The proposed study	Representative previous studies	Scientific merits of the proposed system	References
1. Research integration	Integration of DEP trapping and electroporation modeling in a single COMSOL multiphysics platform	Prior work examined either DEP separation or electroporation independently	Provides a unified computational framework capturing both phenomena simultaneously for therapeutic analysis	^22,54–56^
2. Cell type coverage	Comparative analysis of CTCs, WBCs, and platelets	Previous studies mainly addressed CTC–WBC or CTC–RBC interactions; platelets often omitted	Inclusion of platelets addresses a literature gap, enabling modeling of full-blood components	^44,57^
3. Methodological approach	Dynamic simulation of pore formation, growth, and resealing under 2 $\upmu$s pulses.	Static DEP studies with limited membrane dynamics modeling.	Predicts reversible versus irreversible electroporation through time-resolved membrane kinetics	^22,44,58,59^
4. Electric field optimization	Cell-specific pulse parameters: CTCs/WBCs (1–4 kV/cm), PLTs (10–40 kV/cm).	Generic frequency and voltage ranges without electroporation pulse optimization	Enables selective membrane permeabilization tailored to cell type	^54,55^
5. Parameter completeness	Comprehensive reporting of amplitude, frequency, waveform, pulse duration, and electroporation thresholds	Prior studies reported partial parameters (voltage and/or frequency only)	Supports reproducibility and translational potential	^54–56^
6. Key measurements	Quantification of transmembrane potential, pore size and density, membrane conductivity, and field distribution	Focus largely on separation efficiency; electro-kinetic cell properties not measured.	Establishes a multi-dimensional electrical model linking DEP forces and electroporation	^60,61^
7. Pore dynamics	Temporal tracking of pore formation and resealing.	Cell viability assessed post-treatment; no kinetic modeling.	Predictive model defines thresholds for reversible and irreversible electroporation.	^54–56^
8. Therapeutic control	Tunable reversible and irreversible electroporation via field strength and pulse width.	Previous systems reported binary cell capture outcomes without therapeutic modulation.	Enables controlled drug delivery or gene transfer	^21,54,56^
9. Platform design	Single integrated microfluidic system combining DEP and electroporation	Separate devices utilized in prior studies	Reduces contamination risk and experimental time	^57,62^
10. Membrane resealing	Demonstrates effective resealing after 2 $\upmu$s pulses at $\ge$2 kV/cm	Recovery rarely evaluated	Maintains viability for reversible applications	^54,56^
11. Electric field distribution	Non-uniform field aligned with DEP trapping pattern	Uniform or idealized fields assumed previously	Spatial optimization enhances targeting precision	^56,59,63^
12. Selectivity mechanism	Leverages cell-specific dielectric contrasts (conductivity, permittivity)	Prior DEP studies lacked electroporation coupling	Enhanced CTC selectivity through combined DEP-electroporation effects	^44,54,60^
13. Simulation validation	Full COMSOL multiphysics including thermal, fluidic, and electrical domains	Single-physics or unvalidated simulations in prior work	Predictive capability validated for experimental implementation	^58,59^
14. Clinical applicability	Quantified parameters support direct translation to clinical protocols	Previous studies provided proof-of-concept only, with limited scalability	Bridges computational predictions and experimental design for clinical use	^21,54,57^
15. Biological variability	Accounts for dielectric heterogeneity among blood cells	Limited sensitivity analysis in prior studies	Improves prediction of patient-specific responses	^44,56,60^
16. Thermal effects	Includes Joule heating with safety assessment	Few studies addressed thermal effects	Ensures operation within safe thermal limits	^53,61,64^
17. Throughput and scalability	Flow-optimized design suitable for clinical-scale samples	Trade-offs between selectivity and throughput in prior systems	Balances efficiency with cell viability	^56,65^
18. Viability preservation	Controlled reversible electroporation maintains cell viability	Viability rarely quantified or optimized	Supports downstream functional assays	^56,57,65^
19. Electrode design	3D sidewall/interdigitated hybrid electrodes for combined DEP and electroporation	Previous electrodes optimized for DEP only	Dual-function design improves capture and treatment efficacy	^55,56,63^
20. Frequency response	Simultaneous analysis of DEP and electroporation frequency dependence	DEP frequency tuning only in prior studies	Enables integrated frequency–amplitude modeling	^60^

## Conclusion and remark

This study presented a comprehensive computational investigation of the integration of dielectrophoresis (DEP) and electroporation within a unified microfluidic platform for the selective manipulation and controlled treatment of circulating tumor cells (CTCs), white blood cells (WBCs), and platelets (PLTs). The DEP mechanism ensures stable spatial confinement of target cells between electrode pairs, thereby enabling precise and reproducible exposure to pulsed electric fields while minimizing adverse thermal and hydrodynamic perturbations. This controlled positioning substantially reduces off-target effects and enhances the overall efficacy and selectivity of the electroporation process.

Cell-type-specific electric pulse parameters were systematically derived to match the characteristic membrane charging times of CTCs, WBCs, and PLTs. This parametric optimization facilitates the transient formation of nanoscale pores in the cell membrane, enabling molecular transport into the cytoplasm without compromising cell viability. The selection of a 2 µs pulse duration was informed by prior electroporation studies demonstrating that microsecond-scale pulses at appropriately calibrated electric field strengths are sufficient for effective membrane charging and pore nucleation while maintaining thermal effects within biologically safe limits^53^. The simulation results demonstrate that the electroporation outcome is governed by the time-dependent interplay between the applied electric field strength and the induced transmembrane potential. Higher field strengths produce more rapid and pronounced membrane permeabilization followed by effective resealing upon pulse termination, whereas moderate field strengths maintain the electroporation response within the reversible and biocompatible regime.

The computational analysis further reveals spatially heterogeneous pore dynamics and electric displacement field distributions across the cell membrane, with the most pronounced effects concentrated at the hyperpolarized pole facing the electrode. These local asymmetries underscore the critical role of cell geometry and the spatial distribution of the applied electric field in determining electroporation outcomes at the single-cell level. The observed penetration of the electric field into intracellular regions suggests the potential for influencing subcellular structures, offering mechanistic insight into the pathways of intracellular molecular delivery.

As summarized in Table 2, the key electroporation and dielectrophoresis parameters were quantified for each cell type under pulsed electric field excitation. The results confirm that a 2 µs pulse duration is sufficient to achieve complete membrane charging while minimizing Joule heating, and that the requisite electric field strength must be tailored to cell dimensions, with 1–4 kV/cm being appropriate for CTCs and WBCs and 10–40 kV/cm required for platelets owing to their substantially smaller diameter and correspondingly higher membrane charging threshold. The transmembrane potential dynamics exhibit rapid polarization at selective membrane poles, with the maximum potential attained at approximately 2 µs. Pore formation and resealing are demonstrated to be dynamic and field-dependent processes, and the investigated parameter space supports efficient reversible electroporation for all three cell types. Membrane conductivity and electric displacement field magnitude increase monotonically with applied field strength, providing quantitative characterization of electroporation thresholds and field–membrane coupling.

Table 3 presents a systematic comparative assessment of the present integrated DEP–electroporation framework against representative prior studies, highlighting the principal contributions of this work. These include the comparative multi-cell-type modeling of CTCs, WBCs, and platelets within a single computational domain; dynamic simulation of pore nucleation, expansion, and resealing kinetics; cell-specific electric field and frequency optimization; incorporation of Joule heating analysis and multiphysics coupling; and device-level integration of DEP trapping and electroporation functionalities within a unified electrode architecture. Collectively, these advances bridge computational predictions with translational microfluidic device design and provide quantitative guidance for future experimental implementation and clinical protocol development.

Notwithstanding the contributions outlined above, several limitations of the present computational framework should be acknowledged. The model assumes a spherical cell geometry for all three cell types, which, while widely adopted in analytical and numerical electroporation studies and well justified for suspended CTCs and WBCs, represents a simplification for platelets, which exhibit a discoid morphology under resting physiological conditions. The spherical approximation may influence the predicted transmembrane potential distribution, pore density localization, and the critical field strength required for electroporation onset, particularly for PLTs where the actual surface curvature and membrane area differ appreciably from those of a sphere of equivalent volume. Future refinements of the model should incorporate realistic, non-spherical cell geometries derived from morphological data to assess the sensitivity of the electroporation predictions to shape-dependent effects. Additionally, the current model employs representative dielectric property values for each cell type and does not account for the stochastic biological variability inherent in patient-derived cell populations. Incorporation of population-level property distributions in subsequent studies would enhance the clinical predictive capability of the framework. Furthermore, while the present work includes an analytical estimation of Joule heating effects, full spatiotemporal thermal modeling coupled with the electroporation dynamics remains a target for future development.

In summary, the results of this study demonstrate that DEP-based cell trapping and optimized pulsed electroporation operate synergistically to provide a safe, effective, and non-invasive approach to the controlled manipulation and treatment of heterogeneous blood cell populations. The integrated computational framework establishes a quantitative foundation for the design of microfluidic bioelectric platforms with applications in cancer diagnostics, immunotherapy, targeted drug delivery, and regenerative medicine. Its capacity to precisely modulate cellular membrane biophysics and enhance targeted molecular transport positions this approach as a promising pathway toward improved regulation and increased efficacy of bioelectric therapeutic interventions.

## Supplementary Information

Supplementary Information.

## Data Availability

The datasets and materials generated or analyzed during the current study are available from the corresponding author upon reasonable request. All relevant data have been included in the manuscript and supplementary materials to ensure transparency and reproducibility.
