# Supplementary material for: Integrated electrical modeling of circulating tumor cells for enhanced dielectrophoretic trapping and electroporation
Source: Sci Rep. 2026 Apr 12;16:12072. doi: 10.1038/s41598-026-45747-z (PMC13077043; doi:10.1038/s41598-026-45747-z)
Supplement: Supplementary file 1 — Supplementary Information. [file 41598_2026_45747_MOESM1_ESM.docx]

**Electric Displacement Field Analysis Across Multiple Field Strengths**

This supplementary analysis presents a comprehensive series of contour plots (Figs. S1–S12) illustrating the electric displacement field norm (V/cm ) distribution across circulating tumor cells (CTCs) following electric field pulse application; each figure visualizes polarization effects and spatial transitions at distinct field strengths and time points.


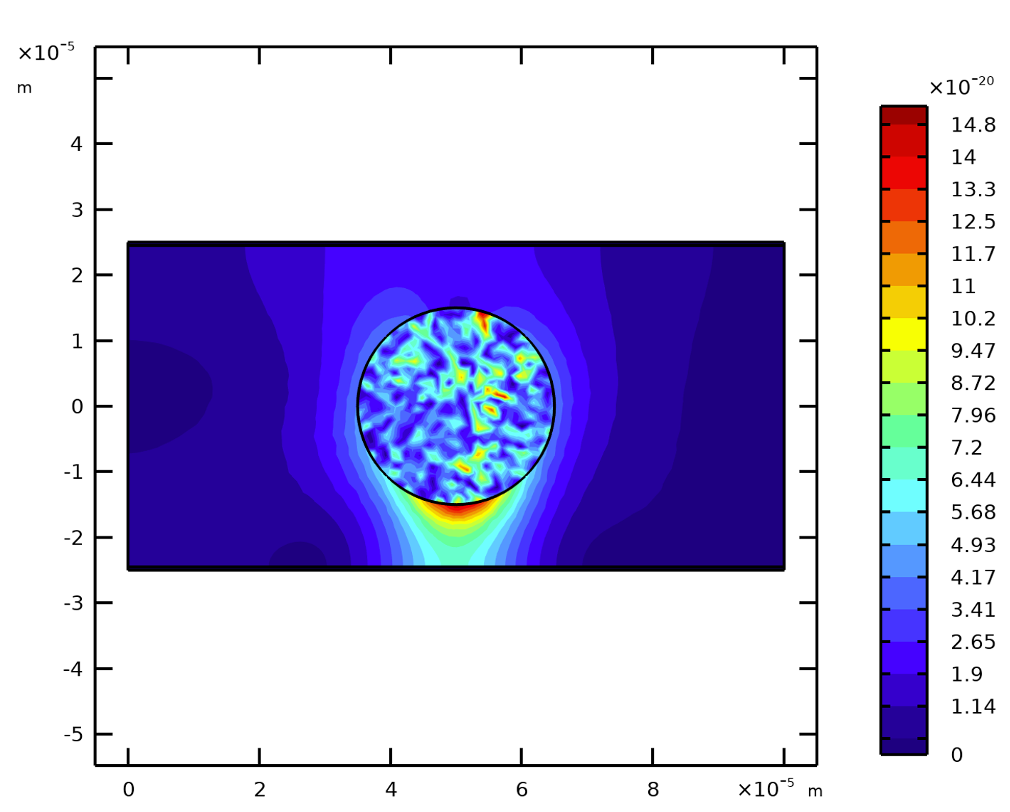


Fig. S1. Electric field displacement across the cell at 20 seconds following a 2µs pulse of 1000 V/cm; color gradients illustrate hyperpolarization and depolarization pole, with clear distinction between interior and exterior field distribution.


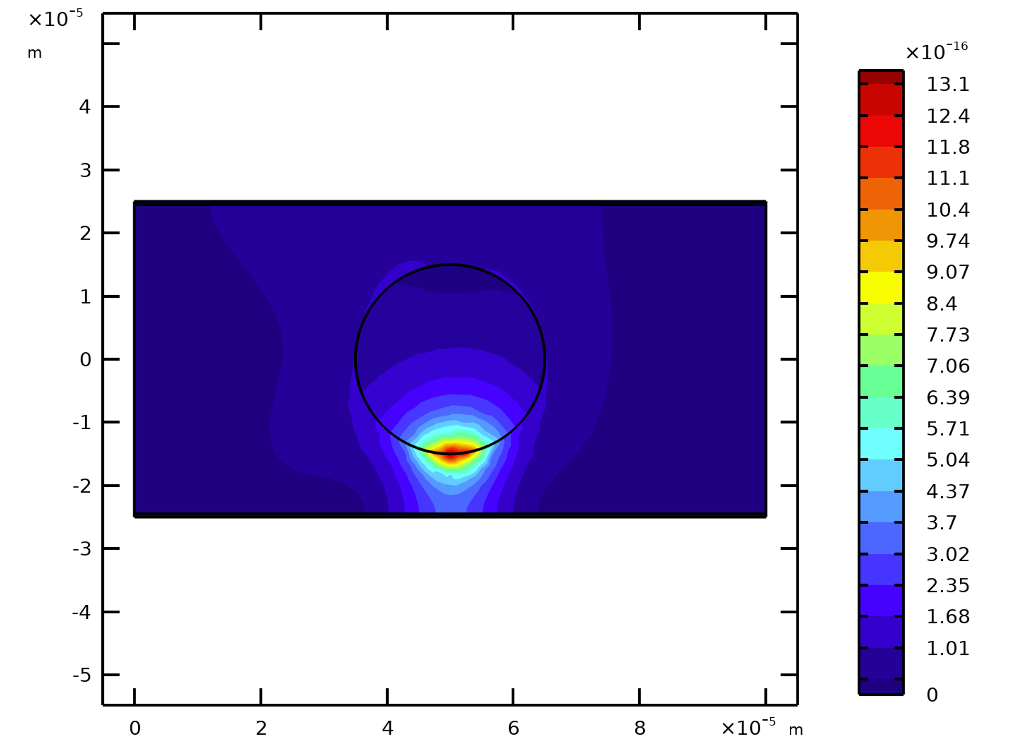


Fig. S2. Electric displacement field distribution for CTC cells at 20 seconds following a 2 µs pulse with an applied electric field of 2000 V/cm, illustrating the spatial polarization and gradient flow across the cell membrane


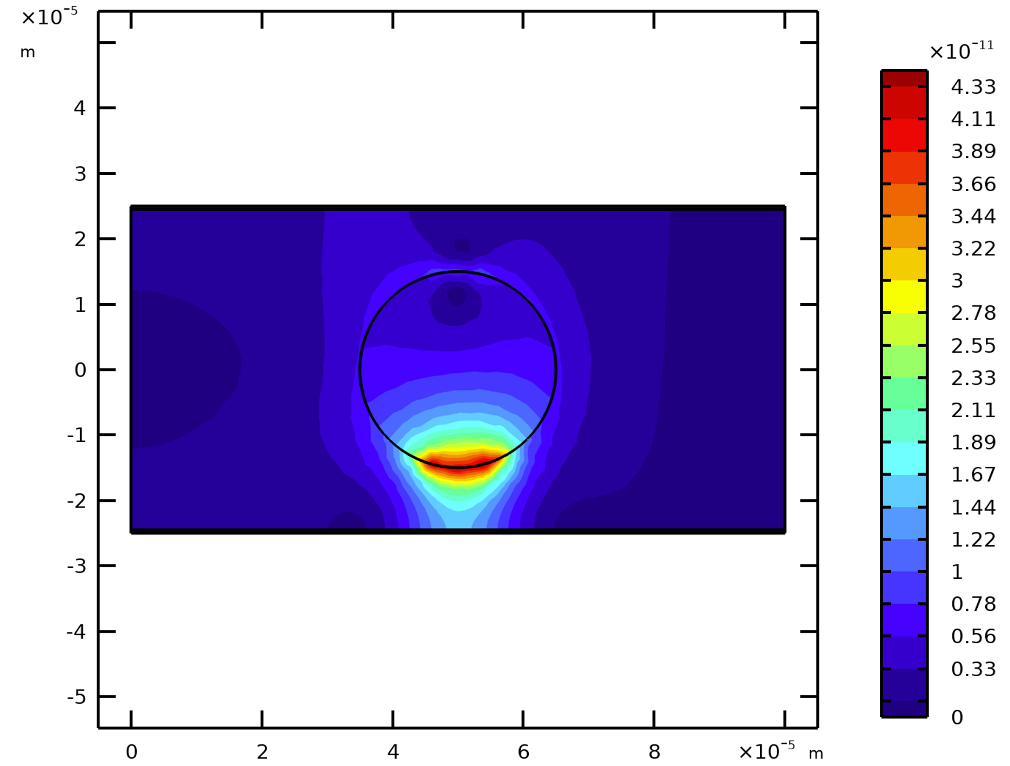


Fig. S3. Electric displacement field distribution at 20 seconds following a 2 µs pulse with an applied electric field of 3000 V/cm in CTC cells


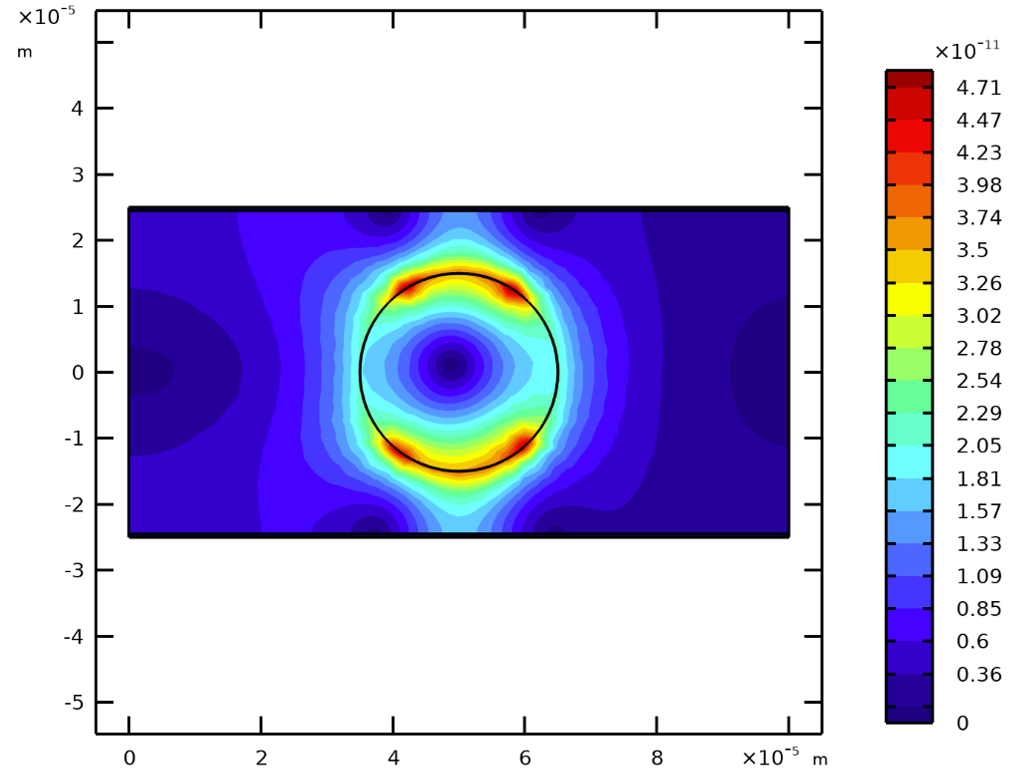


Fig. S4. Electric displacement field distribution with an applied electric field of 4000 V/cm in CTC cells


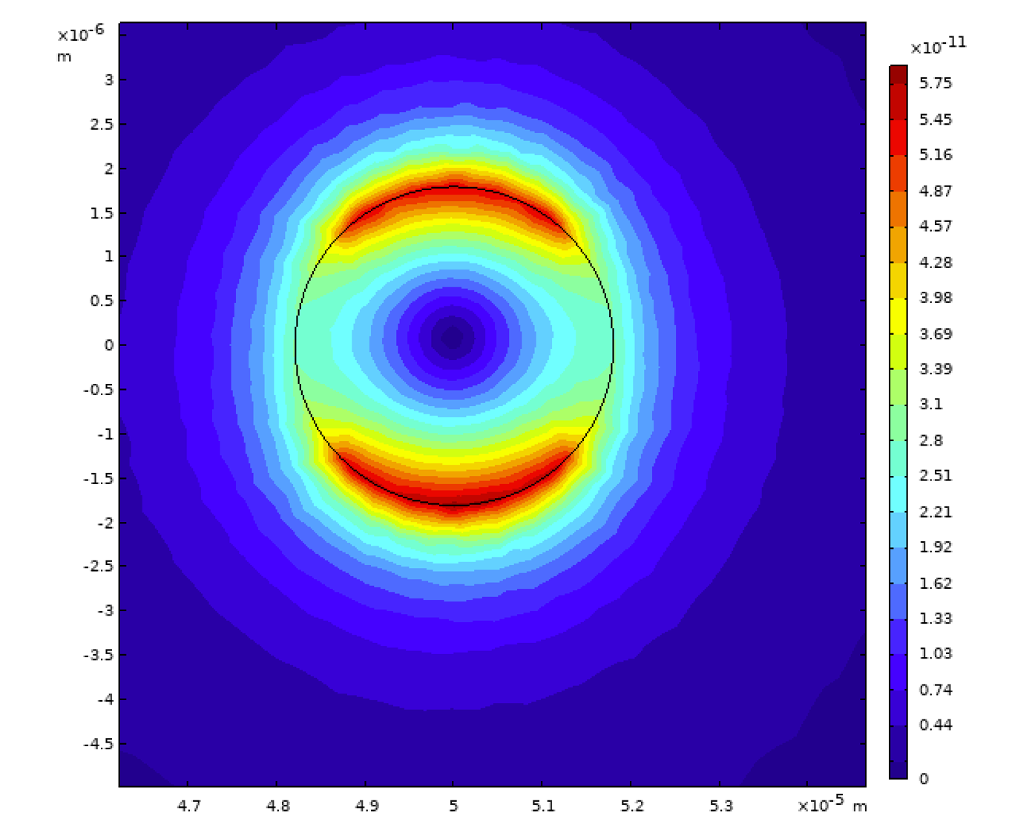


Fig. S5. Electric displacement field distribution in PLT cells under a 10 kV/cm electric field.


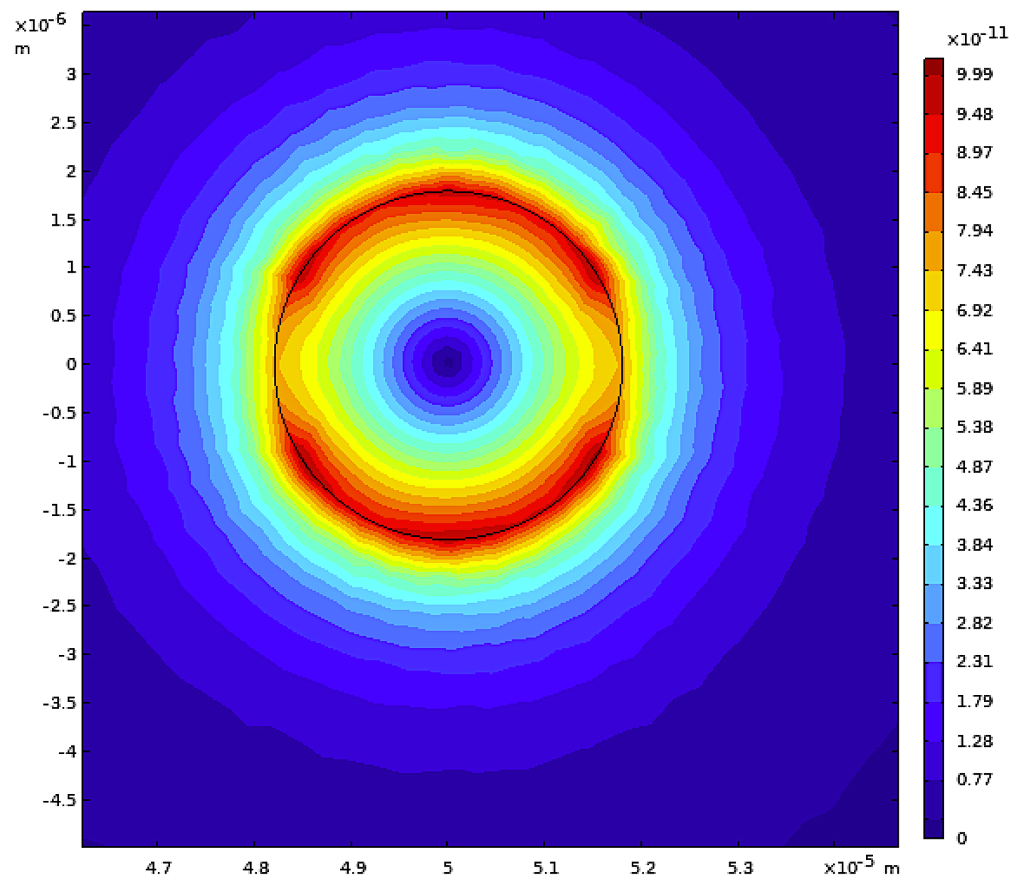


Fig S6. Electric displacement field distribution for PLT cells exposed to an electric field of 20 kV/cm.


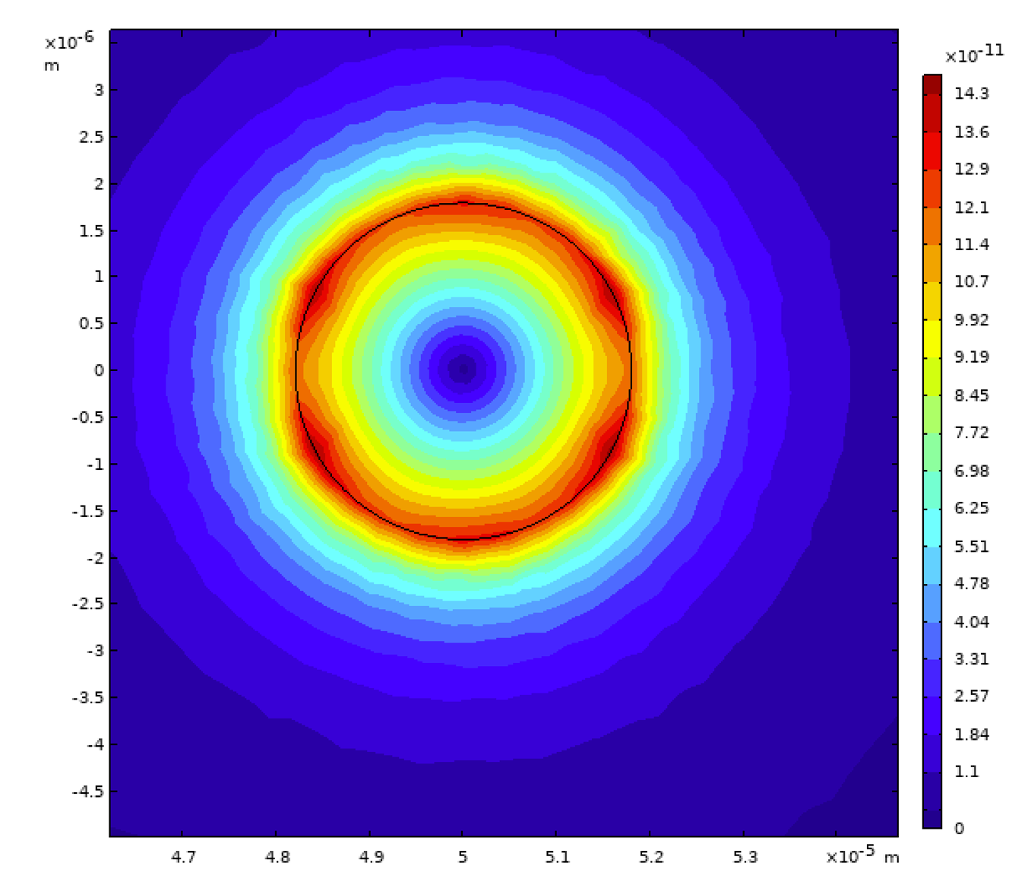


Fig S7. Electric displacement field distribution for PLT cells exposed to an electric field of 30 kV/cm.


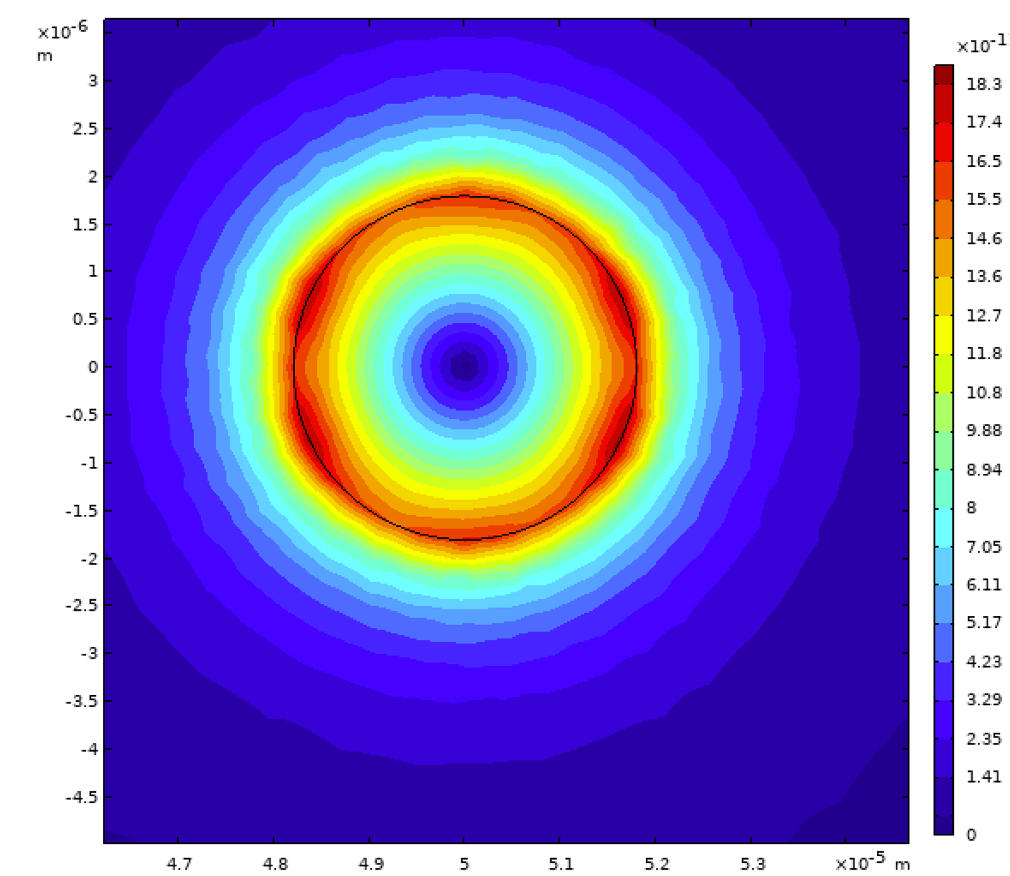

Fig S8. Electric displacement field distribution for PLT cells exposed to an electric field of 40 kV/cm.


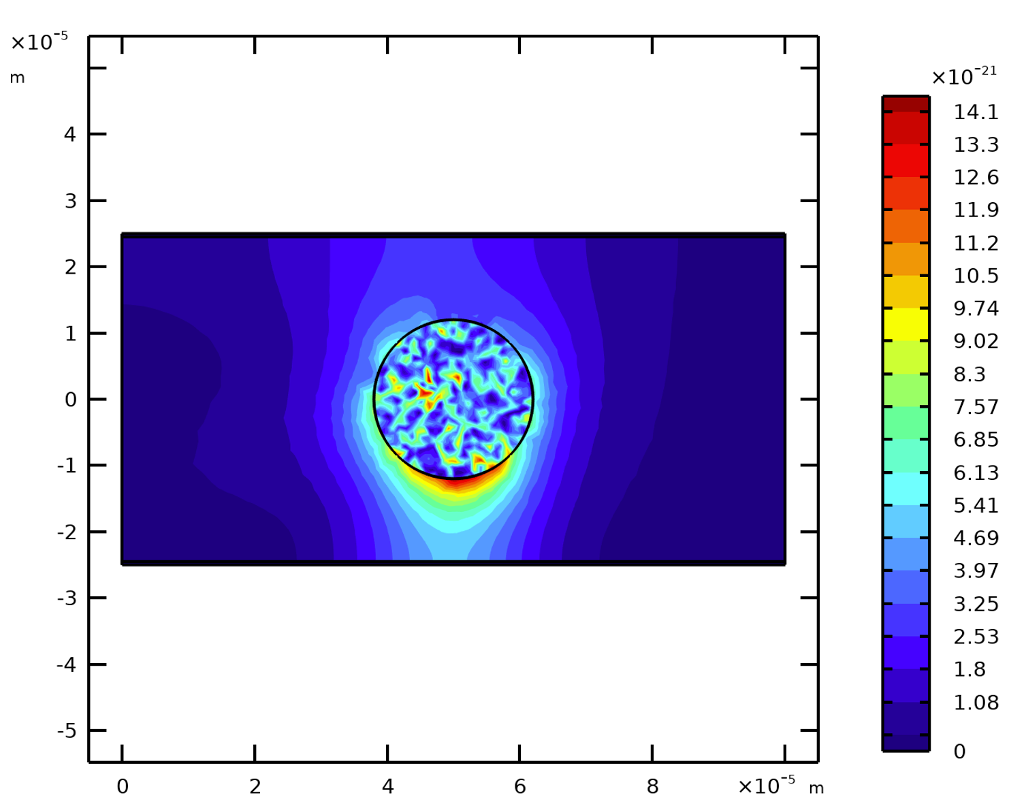

Fig S9. Electric displacement field distribution in WBC cells under an applied electric field of 1 kV/cm.


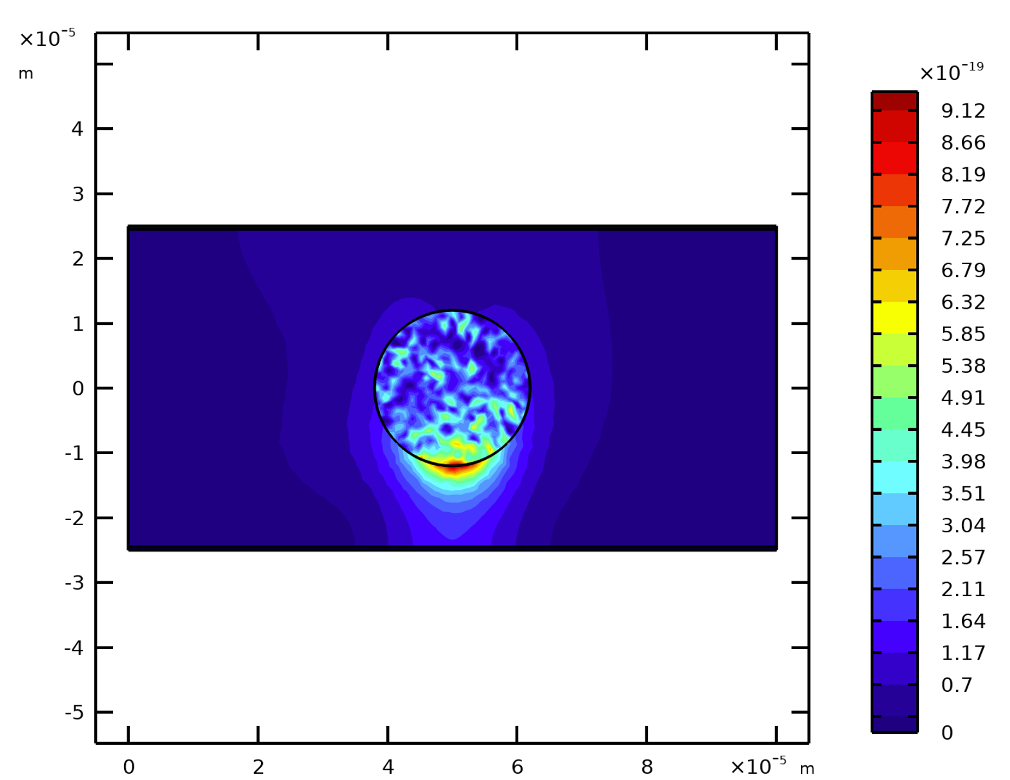

Fig S10. Electric displacement field distribution in WBC cells under an applied electric field of 2 kV/cm.


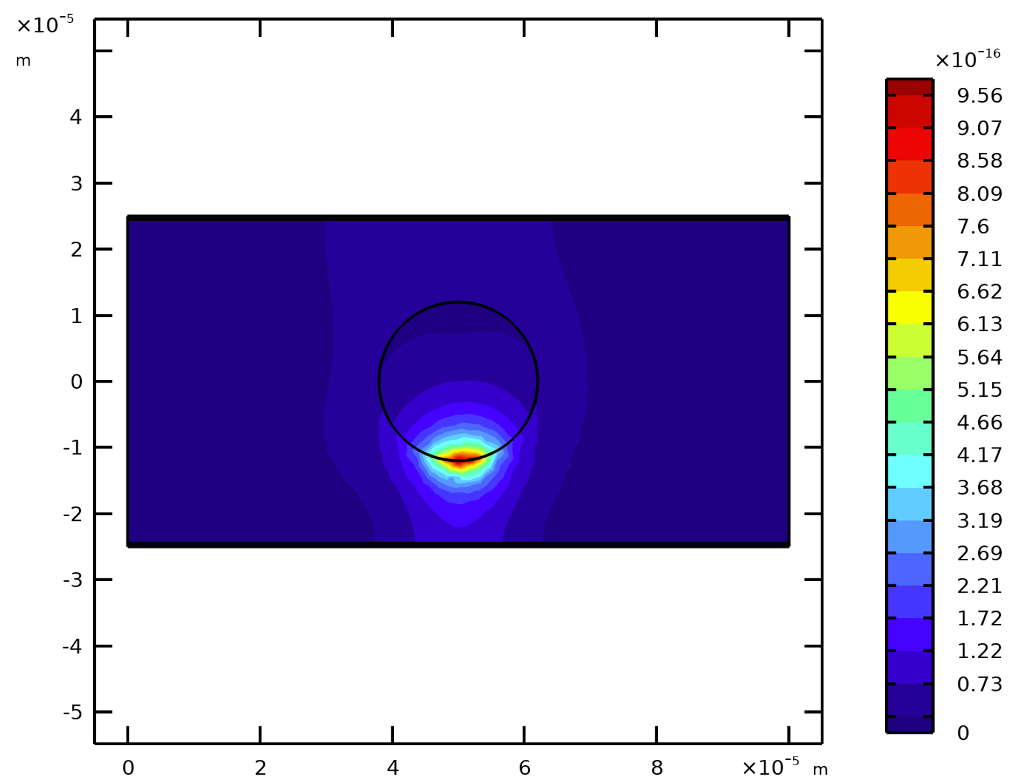


Fig S11. Electric displacement field distribution in WBC cells under an applied electric field of 3 kV/cm.


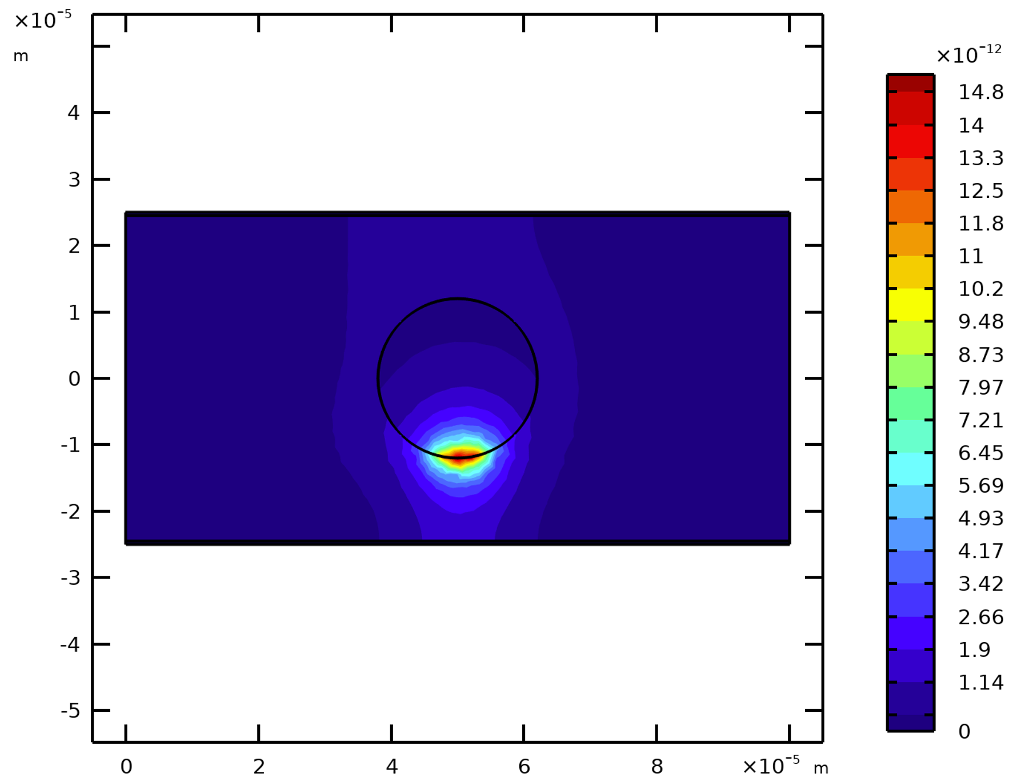


Fig S12. Electric displacement field distribution in WBC cells under an applied electric field of 4 kV/cm.


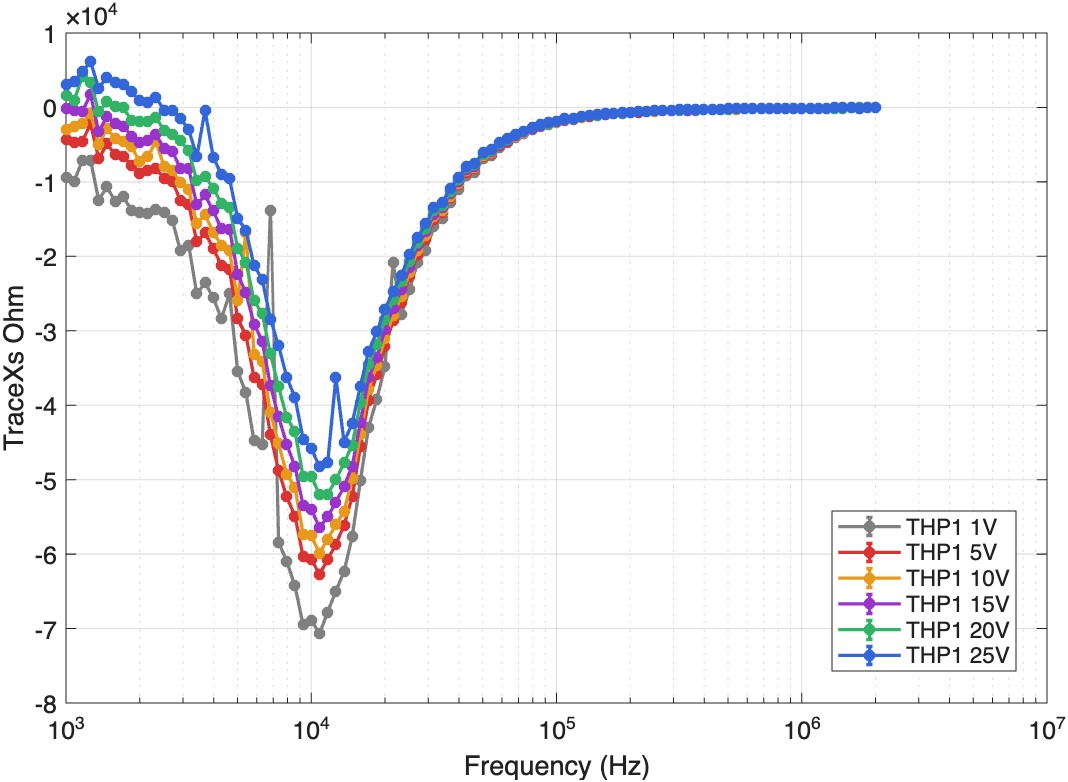


**Figure S13. Voltage‑dependent reactive impedance (Xs) spectra of THP‑1 cells.**
Series reactance measured from ${10}^{3}$to ${10}^{6}$Hz under baseline conditions (1 V) and following electroporation at 5–25 V. The y‑axis is scaled by ${10}^{4}$Ω. Baseline cells exhibit strongly negative reactance at low frequencies, consistent with intact membrane capacitance. Increasing voltage produces a progressive reduction in the magnitude of negative Xs, with near‑complete suppression at 20–25 V, indicating substantial loss of capacitive membrane behavior.


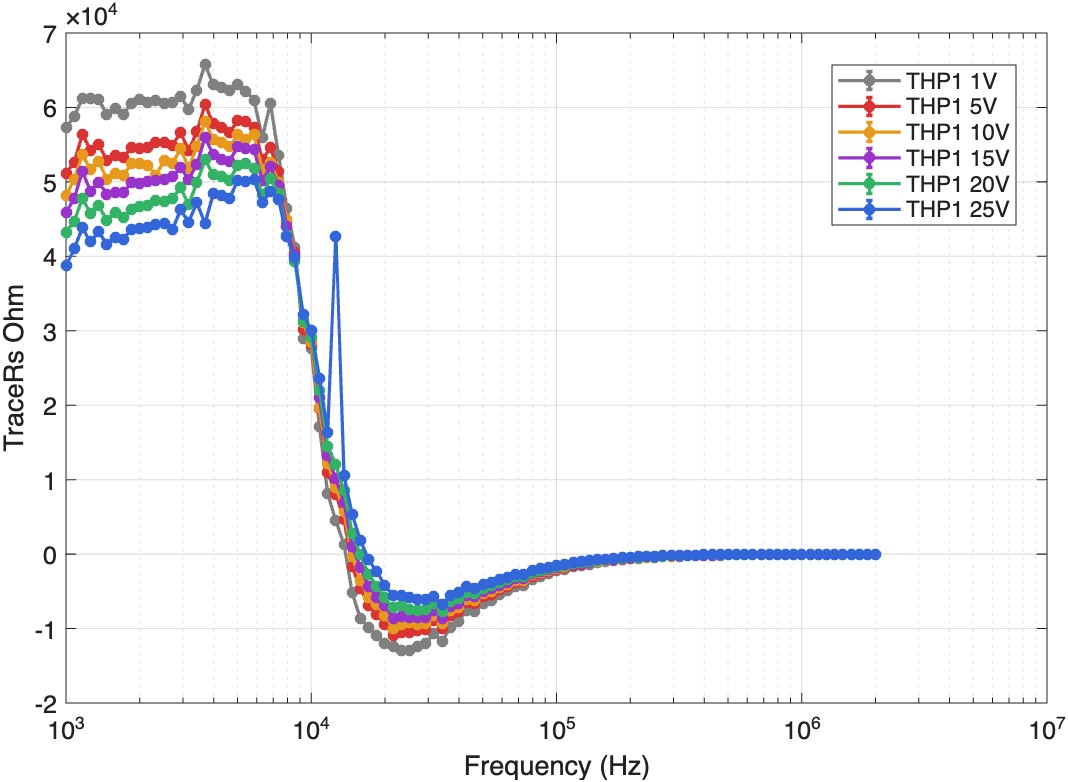


**Figure S14. Voltage‑dependent series resistance (Rs) spectra of THP‑1 cells.**
Real impedance measured from ${10}^{3}$to ${10}^{6}$Hz under baseline (1 V) and 5–25 V electroporation conditions (y‑axis scaled by ${10}^{4}$Ω). The baseline trace shows the largest low‑frequency variability, including negative excursions due to capacitive phase effects.
